# Sea Cucumber *Holothuria glaberrima* High-Quality Genome: new and extended gene families in Holothuroidea

**DOI:** 10.21203/rs.3.rs-9490613/v1

**Published:** 2026-05-20

**Authors:** Joshua G. Medina-Feliciano, Joseph F. Ryan, Michael Grapin, Julianys Tirado Alicea, Amanda Hernandez-Perez, José E. García-Arrarás

**Affiliations:** 1 University of Puerto Rico at Río Piedras, San Juan, PR, United States;; 2 Whitney Laboratory of Marine Bioscience, University of Florida, St. Augustine, FL United States;; 3 University of Florida, Department of Biology, Gainesville, FL, United States;; 4 Juniata College, Huntingdon, PA, United States

## Abstract

The sea cucumber *Holothuria glaberrima* is a premier model for regenerative research that has allowed the discovery of key cellular and molecular characteristics of the intestinal and neuronal regeneration. However, the lack of a high-quality reference genome has limited molecular studies of regenerative processes in this species. We generated a chromosome-level genome assembly using PacBio long-read sequencing and Omni-C chromatin conformation capture data. In addition, assembly improvements were performed through manual curation and scaffolding with artificial mate-pairs from the short read draft genome assembly. The final assembly comprises 1.23 Gb with N50 of 50.9 Mb and 94% of genomic content within 23 chromosomal scaffolds. We identified 34,720 protein-coding genes and evaluated its quality through the analysis of the highly conserved gene families of Hox and Sox genes. In depth genomic analyses showed notable gene family expansions such as 51 THAP domain transcription factors and 37 SRCR domain-containing genes, representing significant increases compared to vertebrates. Furthermore, transposable elements annotation showed that these constitute ~50% of *H. glaberrima’s* genome, with DNA transposons predominating over retrotransposons. Analysis of Gypsy retrotransposons showed upregulation during distinct stages of intestinal regeneration, with expression correlating to nearby regeneration-associated genes. This chromosome-level assembly enables sophisticated molecular studies of regenerative processes and establishes a framework for comparative genomic analyses with deuterostomes and other model species, significantly enhancing our understanding of the genetic basis underlying exceptional regenerative capacity in echinoderms.

## Introduction

**Figure 1.**
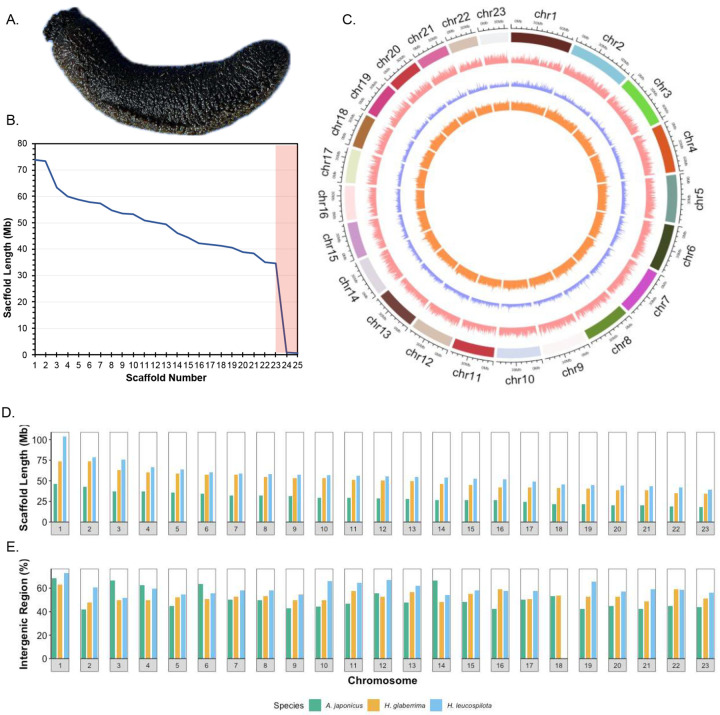
The genome assembly of the sea cucumber *H. glaberrima* 23 chromosomal scaffolds. (A) Image of the sea cucumber *H. glaberrima*. (B) Length distribution of the longest 25 scaffolds of the assembled genome of *H. glaberrima*. (C) Circle plot of the sea cucumber 23 chromosomal scaffolds from the outside to the inside representation of the scaffold length, gene density, retrotransposon density and DNA transposon density. (D) Scaffold length in mega bases pairs of the main scaffolds of the assemblies of the three sea cucumber species *H. glaberrima, H. leucospilota*, and *A. japonicus*. (E) Intergenic region percentage per scaffold of each of the species mentioned above. Intergenenic regions were calculated based on the total where no gene structures were present.

Echinoderms comprise one of the largest phyla of organisms, with members present in almost every marine environmental niche. As model organisms, they have been instrumental in advancing our knowledge of biological processes, particularly those related to reproduction, development, and regeneration. Among echinoderms, sea cucumbers are of particular interest. Economically, they have a high value in the Asian aquaculture marker. Scientifically, their potential as models for biomedical applications, especially in regenerative biology, is already advancing our understanding and holds promise for even greater breakthroughs [1–5]. Two species of sea cucumbers have been the focus of these studies: the Japanese sea cucumber *Apostichopus japonicus*, known for its aquaculture potential, and the brown rock sea cucumber *Holothuria glaberrima* (Figure 1A), which is of interest in regenerative research [5]. *Holothuria glaberrima* is the model organism where the processes underlying regeneration are best understood from cellular and molecular perspective [3, 6, 7].

For many years, significant efforts have been made to increase our knowledge of the genetic components that govern regeneration processes such as proliferation, apoptosis, dedifferentiation, and differentiation, among others crucial for regeneration [5, 7–9]. Consequently, a wide repertoire of gene expression data has been generated for distinct timepoints of the regenerative process in the intestine and radial nerve cord of *H. glaberrima* [10–13]. These data have provided a comprehensive understanding of the determinant genes at different stages of regeneration and the genetic composition of the species. However, the lack of a reference genome has limited our ability to fully leverage newer sequencing techniques that have been continuously developed.

As has been exemplified by the purple sea urchin *Strongylocentrotus purpuratus*, genomic data is needed for the development of new molecular tools for in-depth studies of cellular, developmental and physiological processes. The sea urchin genome assembly allowed the rapid expansion of the molecular knowledge about this species genomic developmental features [14, 15]. Thus, in our case, the availability of a high-quality genome has become crucial to extend our molecular studies of *H. glaberrima* and, in particular, our knowledge of the elements involved in regeneration. We had previously reported the first mitochondrial genome and a draft of the nuclear genome of *H. glaberrima* and demonstrated its utility by assessing the genomic structure of melanotransferrin genes [16]. This genome assembly provided a valuable resource that has promoted the adequate characterization of multiple gene families [16–18]. Nevertheless, this assembly was constructed solely from short reads and is therefore highly fragmented.

Since then, the genomic repertoire of sea cucumber species, in particular, members of the *Holothuria* genus, has continued to grow and increase in quality and contiguity. To mention a few, recently the genome of the sea cucumbers *Holothuria leucospilota* [19]*, Holothuria scabra* [20] and *Holothuria fuscocinerea* [21] have been published, all demonstrating to contain anchored chromosomal scaffolds. In addition, there has been a continuous effort to continue improving the genomic data available for *A. japonicus* with as of this publication four genome being published [22–24]. Several genome assemblies have been reported in recent years for various other sea cucumber species as *Stichopus monotuberculatus* [25] and *Stichopus fusiformiossa* [26], among others. The generation of these genomes has provided not only a valuable perspective on the genomic architecture of the sea cucumber genomes across diverse lineages, but the assembled genomes have been used to answer distinct questions concerning echinoderm physiology and evolution. For example, the genome of *H. leucospilota* allowed the discovery of the molecules that provide the adhesive and matrix nature of the Cuverian tubules of this species [19]. Another example is the description of the molecules involved in saponin biosynthesis, and other features including aestivation and regeneration [23]. In addition, many of these genome studies include the description of conserved gene families as the Hox gene cluster, to display both the quality and the genomic organization of key features [23, 24, 27]. Therefore, it is clear that in order for research on the sea cucumber *H. glaberrima* to advance, it is imperative to generate a high-quality assembly of its genome. Only in this way we will be able to maintain this species as model organism with comprehensive availability of molecular toolbox to decipher the foundations of the organ regeneration process.

Here we report the first high-quality reference genome assembly of the sea cucumber *H. glaberrima*. In addition, we display for the first time the genomic organization of the Hox and Sox genes in this species and highlight their conservation with echinoderms and other species. We further describe many aspects of its genomic composition and organization using distinct genetic features that could provide important and unique information about its regenerative capabilities. Many of these features have arisen due to their upregulation in differential expression data of regenerative timepoints. Others because of the probability of being extended gene families within this species. Among these features, we have included transposable element, focusing on the class II retrotransposons, and two gene families: THAP and Scavenger Receptors Cysteine Rich (SRCR) domains-containing families, respectively.

## Methods

### Sample and Data Collection

A single adult male sea cucumber (Figure 1A) was collected from northeastern Puerto Rico and kept in aerated sea water until dissection. Gonads were collected into ethanol, frozen and sent to Cantata Bio for DNA extraction, library preparation, and sequencing.

### Genome Assembly and Annotation

Initially PacBio CLR sequencing data totaling 155.6 Gbp were assembled with WTDBG2 v2.5 with minimum read length of 20000 and minimum alignment length of 8192 by Dovetail (CantataBio). Output assembly was further improved using Omni-C sequencing data utilizing the HiRise pipeline [28]. Manual corrections were done through breaks created during manual curation of candidate genes based on alignments against *H. glaberrima* draft genome using mummer v4.0.0 [29] and minimap2 v2.22 [30]. A custom script was used to incorporate portions of the genome that were present in the draft genome assembly but absent in the newly assembled genome. After manual corrections, we performed generated artificial mate-pairs from the draft genome assembly using matemaker v1.2 [31] and then scaffolded this assembly using SSPACE v3.0.

Initial gene prediction was performed using BRAKER v3.0.7 [32] hybrid annotation pipeline which incorporates *ab initio* predictions using GeneMark-ETP v1.02 [33] and Augustus v3.5.0 based on *H. glaberrima’*s RNA-seq data and protein hints with ProtHint v2.6.0 metazoan protein database from OrthoDB v11 [34]. To identify the optimal gene models, we utilized the ProtHint and *ab initio* results from BRAKER to obtain a new gene model set with TSEBRA v1.1.2.2. Additional gene evidence and predictions were generated with PASA v2.5.3 [35] using available transcriptome from intestinal and radial nerve tissue RNA-seq of *H. glaberrima*. Protein hints from the closely related species *S. purpuratus* (sp5.0) and *H. leucospilota* (GCA_029531755.1) were generated through miniport v0.12 alignments. All this gene evidence was utilized as input for EVidence Modeler (EVM) v2.1.0 [36] including Augustus and GeneMark-ETP *ab initio* from BRAKER, protein hints from BRAKER and miniport, and PASA assemblies. After evaluation Augustus gene models were selected as the optimal predictions and UTRs were added based on Stringtie v2.2.1 [37] data using the stringtie2utr.py script. Final gene models were then annotated using UniProt/SwissProt complete and human (UP000005640) reference database (date: 01/24/2024). Completeness of each genome assembly and gene model prediction set was assessed with BUSCO v5 [38] with gVolante2 [39].

### Comparative Genomics

We used OrthoFinder v2.5.4 (Emms and Kelly, 2019) with default parameters to generate orthogroups from the protein models of the newly assembled *H. glaberrima* genome and various echinoderm and vertebrates. The following were downloaded from NCBI: *A. japonicus* 2017 (GCA_002754855.1), *Homo sapiens* (GCF_000001405.26), *Mus musculus* (GCF_000001635.26), and *H. leucospilota* (GCA_029531755.1). The protein models from *D. rerio* were obtain from Ensembl (GRCz11), *S. purpuratus* models from Echinobase [40] (sp5.0), the *Ambystoma mexicanum* models were obtain from axolotl-omics.org (AmexT_v47). The latest protein models from *Paracentrotus lividus, H. scabra* and *A. japonicus* were obtained from the data collections documented in the corresponding publication [20, 22].

### Transposable Element Identification and Characterization

Identification and characterization of transposable elements was carried out with two main approaches RepeatMasker v4.1.5 [41] and the Extensive de novo TE Annotator (EDTA) [42]. The RepeatMasker annotation was performed by first creating a repeat sequences database with RepeatModeler v2.0.5 [43], the identified sequences as well as repetitive sequences from RepBase where then utilized to run RepeatMasker. Two distinct versions of EDTA (v2.1.1 and v2.2.1) were utilized to annotate the transposable elements of *H. glaberrima*. The EDTA annotation employs multiple transposable element annotators, many of which are specific for LTR discovery, among these are LTR_FINDER, LTRharvest, LTR_retriever, TIR learner, HelitronScanner, RepeatModeler and RepeatMasker. The initial stage of the annotation was done with EDTA_raw to identify raw transposable elements within the genome and then combined with the full EDTA annotation pipeline to increase the number of intact elements identified. The EDTA v2.2.1 was utilized to identify and annotate the transposable elements of other species whose genome were obtains from NCBI or individual repositories. The following were downloaded from NCBI: *Drosophila melanogaster* (GCF_000001215.4)*, M. musculu*s (GCF_000001635.27), *Xenopus laevis* (GCF_017654675.1)*, Danio rerio* (GCF_000002035.6), and *H. leucospilota* (GCA_029531755.1). The genome of *S. purpuratus* (sp5.0) was obtained from EchinoBase [40] and the genome of *A. japonicus* was obtained from the genome article repository [22]. Results from EDTA were further processes in R for comparisons across species.

### Gene Families Characterization

#### Hox and Sox

Characterization of the *Hox* genes was performed initial identification of the cluster through the annotation with the reference database of Uniprot as mentioned above. Furthermore, the sequences were confirmed as Hox using reference gene sequences of *H. leucospilota* Hox genes from NCBI. Annotation of Hox genes was refined by first identifying Hox transcripts from our transcriptomic data and then utilizing these sequences to confirm or adjust splice junction sites in Hox genomic loci. The sequences utilized for the phylogenetic tree were obtained through BLASTP of *H. glaberrima* Hox genes against EchinoBase and NCBI. In addition, we obtained the sequences from *M. musculus* and *Saccoglossus kowalevskii* from NCBI.

The Sox gene family characterization was done by initially performing a BLASTP of *S. purpuratus* sequences against our transcriptome and predicted gene models. Transcripts were used to confirm the predicted gene models across the genome. These sequences were then utilized to identify other echinoderm Sox genes through BLASTP in EchinoBase or directly against the gene models of the sea cucumber species. The Sox genes from *M. musculus* were obtained from NCBI. The gene tree was generated by constructing a multiple sequence alignment (MSA) of the peptide sequences with MAFFT v7.480 [44] and the phylogenetic tree construction was generated with IQ-TREE v2.3.2 [45] under default parameters.

#### THAP Transcription Factors Gene Family

Initial identification of the transcription factors of *H. glaberrima* and all other species was performed using the cataloguing tool CREPE [46]. The transcriptomic data of each species was obtained from NCBI or EchinoBase. The datasets downloaded from NCBI are the following: *H. sapiens, M. musculus, D. rerio*, *X. laevis, D. melanogaster*, *H. leucospilota*, *Hydra vulgaris* (GCF_038396675.1), *Nematostella vectensis* (GCF_932526225.1), *Caenorhabditis elegans* (GCF_000002985.6), and *Octopus vulgaris* (GCF_006345805.1). The datasets from the following echinoderms were obtained from EchinoBase: *S. purpuratus* (v5.0)*, Patiria miniata* (v3.0)*, Lytechinus variegatus* (v3.0)*, Anneissia japonica* (v1.0)*, Acanthaster planci* (v1.0)*, Lytechinus pictus* (v3.0), and *A. rubens* (v1.3). The datasets from *A. japonicus* and *H. scabra* were obtained from the above-mentioned repository. The presence of the THAP domain sequences identified by CREPE was confirmed with NCBI Conserved Domain Search tool. The THAP and additional domains were characterized based on a cutoff of 1E-05 E-value. From the THAP sequences identified only one isoform from each unique protein model was retained.

#### Scavenger Receptor Cysteine-Rich (SRCR) Domain Containing Gene Family

Genes containing Scavenger Receptor Cysteine-Rich (SRCR) domains were identified from the transcriptomes of *H. glaberrima* by performing BLAST searches using orthologous genes corresponding to Neurotrypsin (NT), Deleted in Malignant Brain Tumor 1 (DMBT1), and representative SRCR-containing genes as queries. The orthologues genes were obtained from the National Center for Biotechnology Information (NCBI) database and consists of NT *S. purpuratus* (XP_030831393.1), SRCR12 *S. purpuratus* (NP_999762.1), DMBT1 *S. purpuratus* (XP_030834721.1), DMBT1 *H. sapiens* (KAI2557538.1), NT *G. gallus* (XP_015143816.1), DMBT1 *M. musculus* (NP_001334561.1), NT *M. musculus*, and SRCR *M. musculus*.

Open reading frame (ORF) completeness assessment of the identified *H. glaberrima* transcripts was assessed using NCBI ORFfinder to classify sequences as partial or complete. Only transcripts containing a complete ORF were retained for downstream analyses. Retained transcripts were subsequently mapped to the *H. glaberrima* genome annotation to confirm correspondence to unique gene loci and to resolve any redundant or overlapping transcript models. Domain architecture analysis was performed on each predicted protein sequence using InterProScan and NCBI Domain Search Tool to characterize the organization of SRCR domain and identify additional functional domains or structural features. For phylogenetic analysis, predicted protein sequences were aligned using MAFFT and maximum likelihood phylogenetic inference was performed using IQ-TREE2 with automatic model selection, and branch support was assessed using 1,000 ultrafast bootstrap replicates. The resulting tree was visualized and annotated using FigTree v1.4. The phylogenetic dataset included *H. glaberrima* SRCR domain-containing sequences alongside orthologues of Neurotrypsin and DMBT1 retrieved from NCBI, as well as top-scoring UniProtKB/Swiss-Prot BLAST hit for each *H. glaberrima* sequence.

## Results

### Genome Assembly and Annotation

DNA from the sperm of one sea cucumber male was isolated and sequenced from which we obtained a total of 7,244,536 reads of PacBio long-read sequencing for a total coverage of approximately 156X. These reads were first utilized to construct a genome assembly with total length of 1.23 Gb with an N50 of 3,224,671 bases. A total of 92,812,137 read pairs were then used for HiRise scaffolding which led to a total of 1,093 joins. This initial assembly resulted in 3,714 scaffolds with an N50 of 50.9 Mb. We improved the contiguity of this assembly by scaffolding it with artificial mate-pairs generated from the published draft genome assembly [16], which resulted in 1,095 joins leading to 2,619 scaffolds. Assembly corrections were done through comparison to the scaffolds of the draft genome assembly (Figure S1). Genome completeness assessment using BUSCO with metazoan library resulted in a total of 92.77% complete + fragmented genes identified (88.47% complete genes) and 90.59% single copy genes (Table 1). Results showed this assembly to have an L90 of 22, however approximately 94% of the total length of the genome was present in the largest 23 scaffolds (Figure 1B–C). These 23 scaffolds showed to be in a range of 73.67 Mb to 34.48 Mb in size with the next scaffold having a length of 57 Kb, a decrease of ~ 98% in size (Figure 1B). In addition, BUSCO assessment of only these 23 scaffolds resulted in similar results with a 92.66% of complete + fragmented genes identified (88.36% complete). Therefore, based on these results and those of other sea cucumber genomes assembly [19, 20, 22, 23], the 23 largest scaffolds of our assembly most likely correspond to the chromosomes of *H. glaberrima* (Figure 1C). In addition, this genome assembly was statistically comparable to the recently reported genomes of other sea cucumbers, particularly the holothuroids *H. leucospilota* and *H. scabra* whose genome assemblies showed an N50 of 56.10 Mb and 53.52 Mb, respectively (Table S1).

To better understand the composition of our assembly, we compared scaffold lengths and intergenic region percentages with those of the main scaffolds of *A. japonicus* and *H. leucospilota* genomes. Results showed that the scaffolds lengths of *H. glaberrima* and *H. leucospilota* differ mostly in their largest scaffold (#1) with approximately 74 Mb and 100 Mb, respectively (Figure 1D). The rest of the 22 chromosomal scaffolds were closer in length. In contrast, the scaffolds of *A. japonicus* were nearly half as long as of those in *H. glaberrima* and *H. leucospilota*(Figure 1D), potentially reflecting genus-specific genome composition given its minimal assembly fragmentation [22]. We further assessed the percentage of intergenic regions to address the probability that the differences in scaffolds length could be due to the length of their intergenic regions. Interestingly, there were minimal differences in intergenic composition between the three species, with scaffold 2 and 10 of *H. glaberrima* and *H. leucospilota* showing the largest deviations (Figure 1E).

To obtain the best possible gene model predictions, we employed different annotation pipelines including BRAKER3 and Evidence Modeler (EVM). The BRAKER3 predictions were performed using all available RNA-seq data from *H. glaberrima*, which includes intestinal and radial nerve cord RNA-seq from normal and regenerating animals [13, 17] and a metazoan orthologs protein data base partition from OrthoDB v.11 [34]. This annotation resulted in 20,121 gene models with mean sequence length of 401 bp (Table 2). While this looked promising, BUSCO assessment of these models resulted in non-optimal scores with 71.80% complete (C) genes and 80.19% complete + partial (C+P) genes against the metazoan database, which are substantially distant from the expected scores compared to the genome assembly BUSCO results (Table 1 and 2). Therefore, we utilized TSEBRA with results of the annotation tools employed by BRAKER3 including protein hints and *ab initio* predictions of GeneMark-ETP and Augustus. This approach improved the BUSCO scores (86.16% C and 93.08 C+P), but the number of gene models identified increased dramatically to 42,217 which to some extent is reflected in the percentage of complete and duplicated BUSCO genes (16.9%) (Table 2). Nevertheless, assessment of the Augustus *ab initio* gene models employed within the BRAKER3 pipeline showed optimal results with a total of 34,720 gene models and 92.56% C+P BUSCO score and minimal duplicated genes (4.3%) (Table 2). Understanding the importance of generating robust gene models that incorporates information from multiple sources we employed EVM with various gene evidence, including gene models from PASA (using transcriptomes from intestinal and nerve RNA-seq data), Transdecoder models, protein alignment of closely related species (*S. purpuratus* and *H. leucospilota*), Augustus and GeneMark predictions. This approach resulted in 30,363 gene models and BUSCO results close to those of Augustus gene models with 84.38% C and 92.14% C+P. Nevertheless, further exploration of these gene models showed genes with erroneous structures, including highly conserved genes like the *Hox* genes (Figure S2). Therefore, the final gene models selected for the rest of our study were from the Augustus predictions, as these are comparable to gene models from *H. leucospilota* (36,089) and *H. scabra* (34,418) and show adequate gene structure of conserved gene families which will be discussed below. Importantly, out of the 34,720 final gene models, 95% were present in the largest 23 scaffolds (33,038).

### Genome Quality Assessment

#### Comparative Genomics

**Figure 2.**
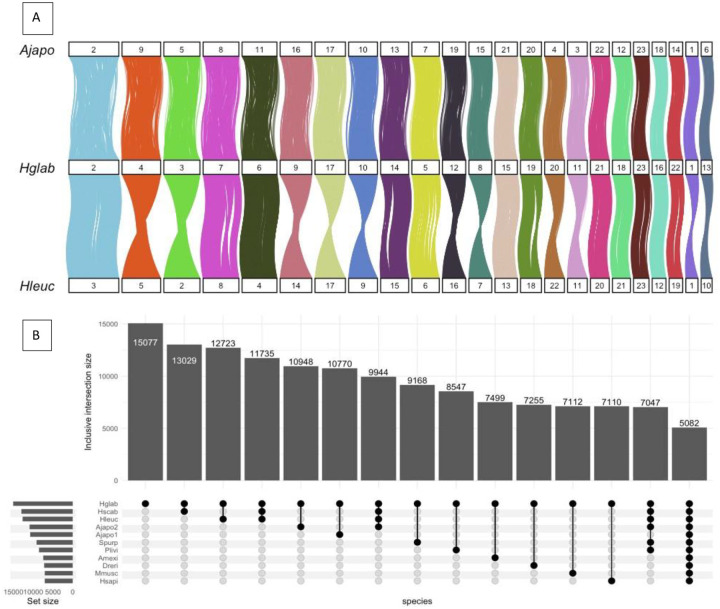
Comparative genomics highlights the quality and fidelity of the genome assembly and gene models of *H. glaberrima*. (A) Macrosynteny plot of *H. glaberrima, H. leucospilota*, and *A. japonicus* chromosomal scaffolds organized by based on the synteny across all species. Plot was generated from single copy orthologs identified with OrthoFinder. Numbered rectangles representing assembled chromosomes are not to scale. (B) Upset plot of the ortholog groups were H. glaberrima overlaps with other echinoderm or vertebrate animals including *H. leucospilota, H. scabra, A. japonicus, S. purpuratus, P. lividus, A. mexicanum, D. rerio, M. musculus*, and *H. sapiens*. Organisms are labeled using the first letter of their genus and 4 letters of the species name. Bars were organized in descending number of ortholog groups shared.

We performed comparative genomic analyses to assess the fidelity of our genome assembly and predicted gene models as well as to identify evolutionary changes at the level of gene conservation and genomic structure. The initial analysis consisted of identifying the orthologs of *H. glaberrima, H. leucospilota, H. scabra* and *A. japonicus* using OrthoFinder. This allowed us to determine the level of conservation of the genome across these species and validate the accuracy of our assembly by observing levels of macrosynteny between these genomes. OrthoFinder results showed a total of 21,667 orthogroups and 7,338 single copy gene orthologs shared across the four sea cucumber species. Visualization the macrosynteny between *H. glaberrima*, *H. leucospilota* and *A. japonicus* based on the single copy gene ortholog groups showed a high genome conservation across their scaffolds (Figure 2A). This analysis showed no evidence of interchromosomal translocations between these 3 species. This macrosyntenic conservation further strengthens confidence in the assemblies of these three genomes. Interestingly, the chromosomal names, which are numbers that reflect the size of the chromosomes (with smaller numbers being larger chromosomes; not to scale in Figure 2A) differ between chromosomal gene linkages. Therefore, despite high levels of conservation in terms of gene content across homologous chromosomes, it appears that the size of chromosomes is dynamic over evolutionary time (Figure 2A). However, this might reflect differences in assembly contiguity of each species. Perhaps the most dramatic differences in scaffold number with synteny between the holothuroids was 9 & 14, and 12 & 16 of *H. glaberrima* and *H. leucospilota*, respectively. The most dramatic difference between the two Holothuria species when compared to *A. japonicus*, was that around 15 out of 23 chromosomal scaffolds showed an offset by more than one chromosomal number (Figure 2A).

We extended our analysis by also identifying the orthogroups shared between *H. glaberrima* and 9 other species including echinoderms and vertebrates: *H. leucospilota, H. scabra, A. japonicus, Paracentrotus lividus* (sea cucumbers)*, Strongylocentrotus purpuratus* (sea urchins)*, Ambystoma mexicanum* (axolotl), *Danio rerio* (zebrafish)*, Mus musculus* (mouse) and *Homo sapiens* (human). Considering the extent to which the initial *A. japonicus* genome has been utilized as a reference for numerous studies we decided to include the gene models of the most recent genome [22] and that of 2017 [24]. We identified 15,077 orthogroups that contained at least one gene of *H. glaberrima* and 5,082 orthogroups shared between all species (Figure 2B). Additionally, we identified 447 *H. glaberrima*-specific orthogroups for a total of 1,469 species-specific genes (Table S2). Furthermore, as expected, *H. glaberrima* shared a higher number of orthogroups with *H. scabra* (13,029) and *H. leucospilota* (12,723), with 11,735 orthogroups containing all three Holothuria species (Figure 2B). From these 11,735 orthogroups, 1,485 were Holothuria-specific orthogroups (Figure 2B). The number of orthogroups shared between *H. glaberrima* and the gene models from the different genomes of *A. japonicus* was similar with 10,948 and 10,770 for the 2023 and 2017 genomes respectively (Figure 2B). In addition, a total of 9,944 orthogroups were identified to contain all four sea cucumber species, with 1,323 being unique to sea cucumber genes (Figure 2B and Figure S3). A total of 9,168 of orthogroups contained genes from *H. glaberrima* and the sea urchin *S. purpuratus*, whereas a 7,047 orthogroups contained all echinoderms species (Figure 2B). Comparisons to the vertebrate species showed a higher number of orthogroups shared between *A. mexicanum* and *H. glaberrima* (7,499), yet the number of orthogroups shared between *H. glaberrima* and *D. rerio, M. musculus and H. sapiens* was still comparable being around 7,000 orthogroups. Furthermore, the species tree generated through this analysis further validates the gene models predicted for *H. glaberrima* (Figure S4). In addition, the high number of orthogroups shared between *H. glaberrima* and the echinoderms, and the constant number of shared orthogroups across vertebrate demonstrate the quality of the gene models generated.

#### Conservation of the Hox gene cluster

**Figure 3.**
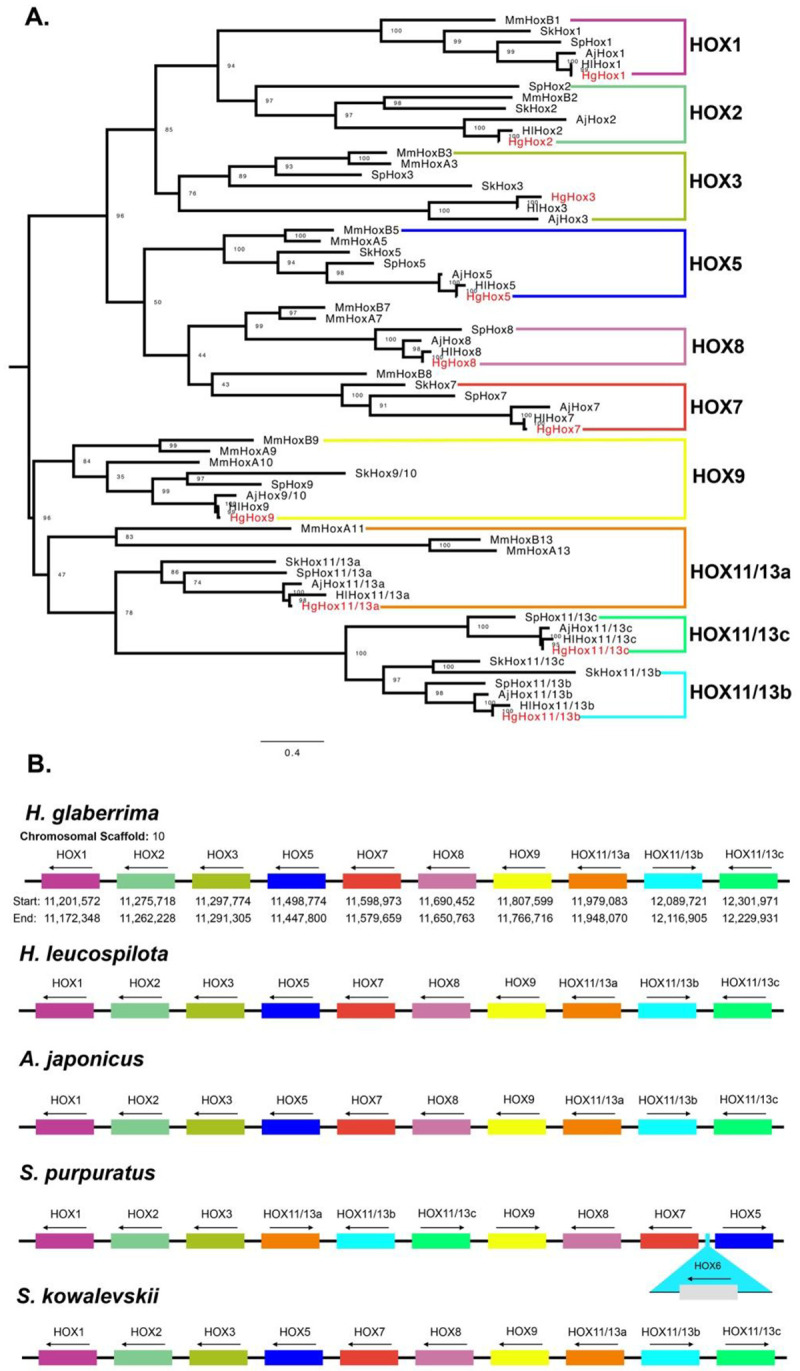
Hox conservation analysis across distinct taxa. (A) Phylogenetic gene tree of Hox genes from distinct species including *H. glaberrima* (Hg), *H. leucospilota* (Hl), *A. japonicus* (Aj), *S. purpuratus* (Sp), *S. kowalevskii* (Sk), and *M. musculus* (Mm). Node labels represent bootstrap values. (B) Genomic organization of Hox gene cluster in the echinoderms *H. glaberrima*, *H. leucospilota*, *A. japonicus*, and *S. purpuratus*, and the hemichordate *S. kowalevskii*. Boxes represent each distinct *Hox* gene. Arrows demonstrate the transcription direction. The genomic range of the gene in *H. glaberrima* are shown under each corresponding *Hox* gene.

Hox genes are important genes in evolution and developmental studies as per their involvement in the body patterning during organism development [47, 48]. Moreover, in recent years they have become important genes for the evaluation of genomic architecture of distinct taxa [49–52]. Therefore, the analysis of these genes and their cluster conservation can be used as a reliable method to evaluate the assembly quality. Here we compare the Hox gene cluster obtained in our *H. glaberrima* genome assembly with that of animals from distinct taxa including *M. musculus, Saccoglossus kowalevskii, S. purpuratus, A. japonicus, H. leucospilota, and H. glaberrima*. Through sequence alignment comparison and gene tree construction our results showed that, as in other echinoderms, the genome of *H. glaberrima* has 10 Hox genes: *Hox1, Hox2, Hox3, Hox5, Hox7, Hox8, Hox9, and Hox11/13a-c* (Figure 3). The gene tree analysis demonstrated the high conservation of these genes between vertebrates and invertebrate species as was expected. Moreover, it confirms our annotation of the Hox genes present in *H. glaberrima*. For instance, the constructed tree showed *M. musculus HoxA9*, *HoxB9* and *HoxA10* to be in the same clade as *Hox9* of the invertebrates in our set. Similarly, *HoxA11*, *HoxA13* and *HoxB13* of *M. musculus* was a sister clade of *Hox11/13a* of these invertebrates. In contrast, The *Hox11*/*13c* and *Hox11/13b* genes of invertebrates formed a monophyletic group without any *M. musculus* Hox gene. As shown in the gene tree of Figure 3A, the distinct Hox genes formed monophyletic groups in accordance with their anterior (*Hox1, Hox2, Hox3*), central (*Hox5, Hox7, Hox8*) and posterior (*Hox 9/10, Hox11/13a, Hox11/13b, and Hox11/13c*) classification.

Genomic characterization of the Hox cluster showed the conserved cluster to be intact in scaffold 10 of our genome assembly (Figure 3B). Furthermore, the order of the identified Hox genes was demonstrated to be maintained between *H. glaberrima, H. leucospilota*, and *A. japonicus*, showing differences compared to other echinoids both in order and absence of *Hox6* (Figure 3B). In sea cucumbers, as in the hemichordate *S. kowalevskii*, all the Hox genes are arranged from Hox1-Hox11/13c in increasing number and in the same direction, except for Hox 11/13b which is in the opposite direction (Figure 3B). This genomic organization is quite distinct to that of the echinoid *S. purpuratus* where the posterior Hox genes appear to be localized adjacent to the anterior Hox genes, which in this species includes *Hox6*. The opposite direction of transcription of the posterior and central Hox genes of *S. purpuratus* has been previously reported to be due to a translocation through inversion of this chromosomal segment [50, 53]. These results further validate the fidelity of our assembly and the high conservation of the genomic architecture of *H. glaberrima* with other sea cucumbers.

#### The Sox genes are conserved across echinoderms

**Figure 4.**
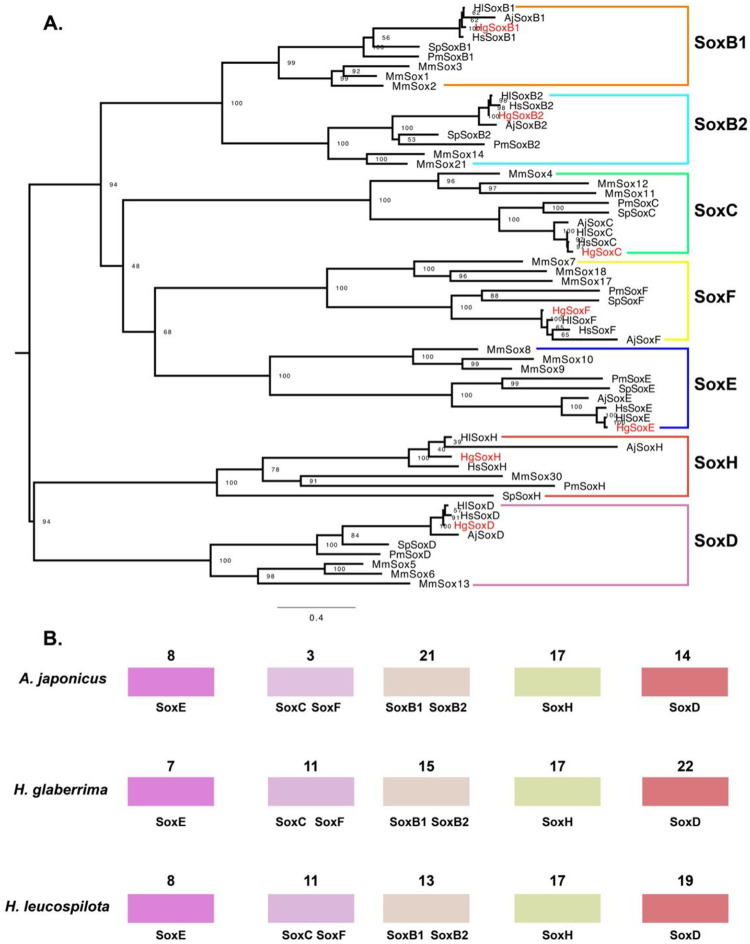
Sox gene tree demonstrates gene family conservation in echinoderms. (A) Gene tree of all the Sox genes from *H. glaberrima, H. leucospilota, H. scabra, A. japonicus, S. purpuratus, P. miniate, and M. musculus*. All the Sox gene sequences were obtained from NCBI. Values in nodes indicate the bootstrap value. (B) Genomic organization of all Sox genes of *H. glaberrima, H. leucospilota* and *A. japonicus*. Rectangles and numbers above correspond to each chromosomal scaffold colored in agreement with the macrosynteny plot of Figure 2A.

To further validate the quality of the genome we assessed the presence of the highly conserved high mobility group (HMG) box domain containing Sox subfamily. This group of genes is highly conserved across species from vertebrates to invertebrates demonstrating similar biological functions across species [54, 55]. Deuterostome Sox genes have been classified into seven distinct clades each containing several Sox gene families [55]. Through sequence similarity search we have identified one member from each of these clades, namely SoxB1, SoxB2, SoxC, SoxD, SoxE, SoxF, and SoxH, which agrees with what has been reported for other echinoderm species [56, 57]. Each Sox gene is named based on the group to which they correspond. To validate the identity of the Sox genes identified in *H. glaberrima*, we performed a phylogenetic analysis that included a range of echinoderm species – three sea cucumber species *A. japonicus, H. leucospilota* and *H. scabra*, the sea star *Patiria miniata* and the sea urchin *S. pupuratus* – and multiple Sox genes of *M. musculus*. Our results showed each Sox gene to be in a monophyletic group per each of the distinct Sox clades present (Figure 4A). Interestingly, *SoxD* and *SoxH* appeared to be in a paraphyletic group as a sister clade to the rest of the classes. In addition, *SoxB1* and *SoxB2* form a sister clade to that of *SoxC*, *SoxF* and *SoxE* (Figure 4A). Notably, in all cases, expect for *SoxH* class, the echinoderms formed a single clade, whereas on all occasions the sea cucumbers are in a paraphyletic group within each of these branches (Figure 4A). In the same way the multiple Sox gene representation per clade of *M. musculus* appeared as sister clade to that of echinoderms (Figure 4A). Thus, Sox genes showed a high sequence conservation throughout all its clades between echinoderms and the vertebrate *M. musculus*.

In addition, we explored the genomic organization of these Sox genes in the sea cucumber *A. japonicus*, *H. lecupospitlota* and *H. glaberrima* to further confirm the synteny across its chromosomal scaffolds (Figure 4B). Here the Sox genes loci agreed with the synteny of the chromosomal scaffolds, with each Sox gene localized in the homologous scaffold of each species. Notably, some of the Sox genes appear to be within the same scaffold across all the sea cucumbers, including *SoxC*-*SoxF* and *SoxB1*-*SoxB2* (Figure 4B). Compared to the monophyletic group *SoxB1* and *SoxB2*, the genes *SoxC* and *SoxF* appear to be polyphyletic (Figure 4A). Interestingly, the discrepancy in chromosomal scaffold can be visualized in detail as *SoxB1* and *SoxB2* appear to be in chromosomal scaffold 21 of *A. japonicus*, but in the chromosomal scaffold 15 and 13 of *H. glaberrima* and *H. leucospilota* respectively (Figure 4B). Another example is that of *SoxC* and *SoxF* localization in chromosomal scaffold 3 of *A. japonicus*, but in the chromosomal scaffold 11 in the two holothuroids (Figure 4B). Thus, raising the question of the correct organization of these chromosomal scaffolds of these sea cucumbers. Are the differences in chromosomal arrangement of *A. japonicus* species specific or is it due to higher quality of contiguous scaffolding? Further experimentation should be performed to answer this question.

### Trasnposable elements in *H. glaberrima*

TEs can comprise a substantial portion of animal genomes, where retrotransposons have been shown to be the most abundant in many eukaryotic genomes. In most mammals, TEs compose about half of the genome, while in other species such as salamanders, they have been suggested to be one of the reasons for their enormous genome size of ~120 Gb, as that of the Mexican axolotl, *Ambystoma mexicanum* [58]. In fact, the family with the most coverage in the axolotl has been reported to be retrotransposons, particularly LTR elements. In addition, non-LTR retrotransposons are known to be active in humans and appear to constitute a great percentage of the sequences in their genome [59]. To date, limited genomic data have confined *H. glaberrima* TE studies to elements expressed at discrete timepoints, making this one of the first efforts to characterize transposable element compositionat the genomic level. Therefore, as part of our initial genome description we characterize the composition of transposable elements in the genome of *H. glaberrima*.

**Figure 5.**
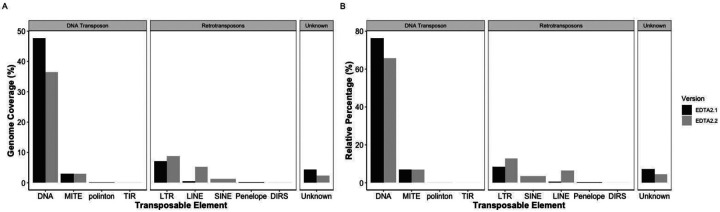
Comparison of genome coverage of the major transposable element classes of *H. glaberrima* annotated with EDTA. (A) Genome coverage of the two classes of transposable elements: retrotransposons (class I) and DNA transposons (class II). (B) Total percentage of elements identified per each class relative to the total elements identified.

The annotation of transposable elements in the genome of *H. glaberrima* resulted in variable family composition comparing two versions of EDTA and RepeatMasker (RM) (Table 3). The overall identification of the transposable elements was the same across them ranging between 48–53% genome coverage (g.c.) (Table 3). Nevertheless, EDTA resulted in better annotation across all families with minimum number of unclassified elements with about 2% g.c. compared to 44% g.c. of unclassified repetitive elements with RepeatMasker (Table 3). The major difference between these two annotation pipelines appeared to be the capability to annotate the DNA transposon class. Out of the total elements identified by RepeatMasker approximately 4% were classified as DNA transposons (2% g.c.), whereas EDTA classified 65% of the total elements as DNA transposons (40% g.c) in v2.1 and 75% (31% g.c.) in v2.2 (Figure 5B). For the retrotransposon class RepeatMasker and EDTA v2.1 annotated a similar percentage of retrotransposons with ~ 9% of total elements (~ 6% g.c.), respectively. Yet, the distribution of this percentage across the different retrotransposon subfamilies varied dramatically with RepeatMasker classifying 6% of the elements as LINE (5.39% g.c.) and EDTA v2.1 0.5% (0.53% g.c.) (Figure 5A–B). Similarly, the percentage of elements identified as LTR element varied even further with 1.2% (0.96% g.c.) vs 8.5% (5.83% g.c.) total elements classified as LTR by RepeatMasker and EDTA v2.1, respectively. Nevertheless, EDTA v2.2 identified a percentage of LINE (6.5% with 4% g.c.) similar to RepeatMasker. This differences in number of elements and percentage of genome coverage were also present between versions of EDTA (Figure 5A–B). Perhaps the most notable difference resulted in the identification of the LINE elements, where EDTA v2.2 showed a larger number of elements for a total genome coverage of 4.20%, compared to 0.53% in EDTA v2.1 (Table 3 and Figure 5A–B). In addition, while the number of LTR elements was higher on EDTA v2.2 it seemed to have decreased the number of Gypsy elements, as 1.22% of elements were annotated as Gypsy compared to 2.19% in EDTA v2.1. Nevertheless, EDTA v2.2 certainly seemed like the optimal tool for the identification and annotation of transposable elements in the genome of *H. glaberrima* as it showed a more diverse categorization of subfamilies and less percentage of unknown elements. Therefore, the annotation from EDTA v2.2 was utilized for further analysis.

**Figure 6.**
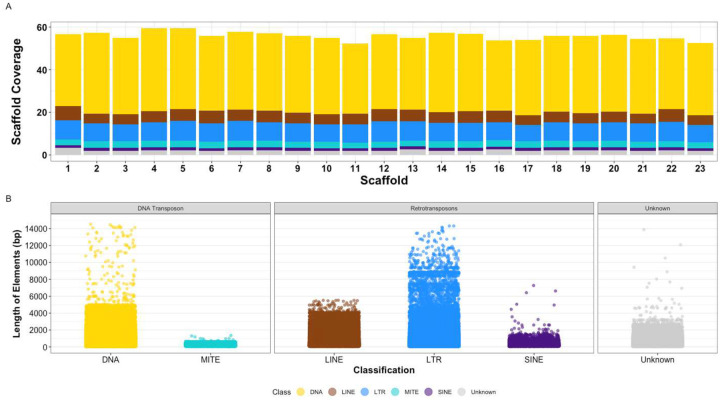
The transposable elements of *H. glaberrima* show constant coverage across scaffolds. (A) Scaffold coverage of the main transposable element classes annotated by EDTA in the genome of *H. glaberrima across* its chromosomal scaffolds. (B) Length distribution of the transposable elements within each main transposable element class.

Both major families (DNA transposons and retrotransposons) have consistent coverage across the 23 chromosomal scaffolds of *H. glaberrima* (Figure 1BC and Figure 6A). Similarly, the density of the elements was also constant across the length of each scaffold (Figure 1C and Figure S5). This suggests that there was no preference of TE insertion to a particular set of scaffolds or locations within them. Additionally, the differences in coverage between the major classes of TE does not seem to be due to the length of elements within each class (Figure 6). The mean length of elements from class I (retrotransposons) and class II (DNA transposons) were comparable at 400 bp and 321 bp, respectively. The length distribution of the DNA transposons and LTR elements varied across subfamilies ranging from 89 (Crypton-A) to 1856 bp (Sola-2) in DNA transposons and 359 bp to 1108 bp (DIRS -- *Dictyostelium* repetitive sequence) (Figure 6B). Comparatively, the LINE elements showed a constant length across subfamilies ranging from 251 bp (CR1-Zenon) to 1057 bp (Jockey) (Figure 6B).

**Figure 7.**
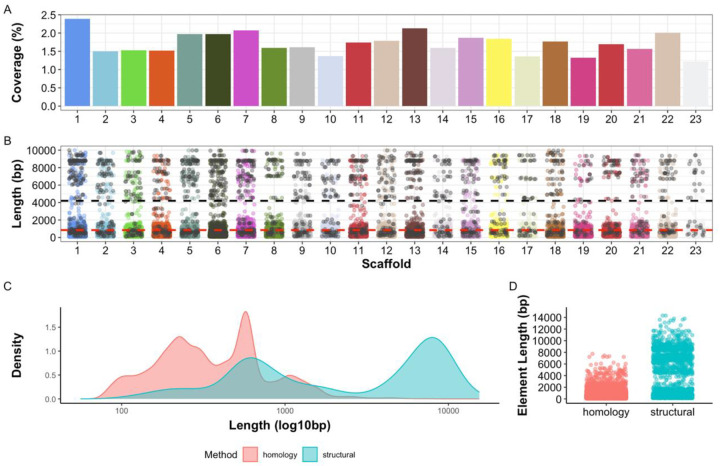
Distribution of the coverage and length of Gypsy elements is constant across scaffolds in *H. glaberrima*. (A) Scaffold coverage of Gypsy elements across the chromosomal scaffolds of *H. glaberrima*. (B) Distribution of Gypsy elemetns lengths across the chormoromal scaffolds of *H. glaberrima*. Black dots = structurally intact Gypsy. Black dashed line = mean length of structurally intact Gypsy. Red dashed line = the mean length of all the annotated Gypsy. (C) Density plot of elements per length classified by their homology or structural identification method. (D) Length distribution of *Gypsy* elements identified by homology or structural method.

RNA sequencing data from our laboratory showed that the *Gypsy* LTR elements were upregulated in the radial nerve complex of *H. glaberrima* during regeneration [60]. In addition, two Gypsy elements (Gypsy1 and Gypsy2) were highlighted as also being expressed at early stages of intestinal regeneration [61]. A possible association between transposons and regeneration has also been hinted at in other regenerating animal species. For example, studies in the Iberian ribbed newt, *Pleurodeles walt*, also showed that specific TEs, including *Gypsy* elements were upregulated during limb regeneration [62]. Yet, until now, the lack of genomic information had limited the ability to further explore the genomic underpinnings of the expression of these elements. Therefore, we further characterized the Gypsy elements to have a better sense of their distribution, location and potential correlation to genes that are upregulated during regeneration. Gypsy elements coverage distribution ranged between 1.2–2.4% across the chromosomal scaffolds of *H. glaberrima*, with minimal differences across scaffolds (Figure 7A). Furthermore, the scaffold coverage did not seem to depend on the length of the scaffold as scaffold 13 had a scaffold coverage of Gypsy elements (2.1%) similar to that of scaffold 1 (2.4%) (Figure 7A). The length distribution across the chromosomal scaffold showed all scaffolds to have elements with lengths over to 4,000 bp, with a range of 56 bp to 15,474 bp (Figure 7B). However, the scaffolds that have the highest coverage do seem to have larger Gypsy elements (Figure 7A–B). This large difference in length distribution of the Gypsy elements appears to reflect the method by which the elements were identified, either by homology to elements or by structural components, with none of the elements identified through homology being larger than 8,000 bp of length (Figure 7C–D). It also demonstrated the importance of having structural based methods to identified retrotransposon elements.

#### Transposable elements and Gene Expression

**Figure 8.**
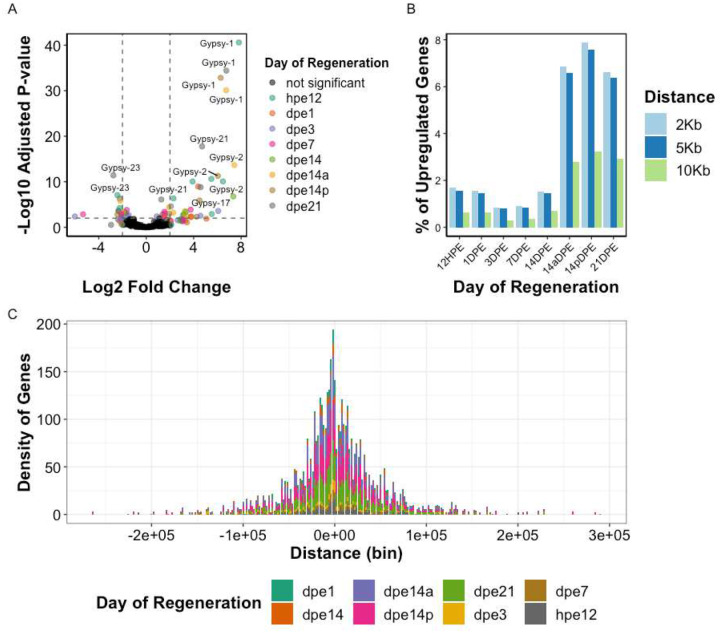
The expression of Gypsy does not seem to be dependent on the expression of nearby genes. (A) Volcano plot of the differential gene expression of *Gypsy* elements at different stages of intestinal regeneration. Black points elements with a padjusted value higher than 0.01 and an expression between −2 and 2 log_2_ fold-change. (B) Bar plot of the percentage of expressed genes out of the total genes within different distances (2Kb, 5Kb, 10Kb) from *Gypsy* elements across the genome of *H. glaberrima*. (C) Density plot of genes upregulated at distinct intestinal regeneration timepoints based on their distance from a *Gypsy* element. Distance bins represent a window size of 2,000 bases upstream and downstream from a *Gypsy*.

Since one of the key interesting aspects of the Gypsy elements is their expression, we wanted to further investigate if this expression is dependent of the expression of nearby genes during regeneration. The specific question that has been poised by previous evaluators of our work was whether the high transposon transcription during regeneration was due to the increase transcription of neighboring genes. We began our analysis by determining the expression of identified Gypsy elements at distinct stages of intestinal regeneration using previously reported RNA sequencing data [13, 17]. To perform this analysis, we utilized the sequences of the Gypsy elements previously published [60] and mapped them to the intestinal regeneration transcriptome to determine their expression across regeneration stages from 12 hours post evisceration (HPE) to 21 days post evisceration (DPE). Results showed that the expression that had been reported in the regenerating nerve tissue for *Gypsy1* and *Gypsy2* is consistent in the regenerating intestine at 12 HPE, 1 DPE, 3DPE, 7DPE, 14DPE and 21DPE (Figure 10A). Many of the other Gypsy elements identified showed to be up- or down-regulated at specific regeneration timepoints including *Gypsy7, Gypsy10, Gypsy17, Gypsy21, Gypsy23, Gypsy25*, and *Gypsy27* (Figure 8A). These results demonstrate that the Gypsy elements have a dynamic expression profile across the regeneration process, which further suggest specificity of transcription during the process.

To assess whether Gypsy element expression is independent of neighboring genes, we analyzed genes located adjacent to EDTA-identified Gypsy elements throughout the genome. This analysis was done by identifying the genes that were within 2Kb, 5Kb, 10Kb and the adjacent gene upstream and downstream of all the Gypsy elements. Then we obtained the percentage of upregulated genes from the total genes identifies within each distance. As expected, we identified a decreasing number of genes as distance was reduced ranging from 14,955 genes within 10Kb to 10,312 genes within 2Kb. Comparatively, we identified 12,498 genes to be adjacent to each Gypsy element identified (the next gene up- and downstream to each Gypsy). From this gene we then determined the total number of genes that had significant upregulation at the different timepoints of regeneration. Two main findings can be gleaned from our results. First, out of the total genes identified per distance the percentage of upregulated genes in the rudimentary/anlage tissue was lower than the percentage upregulated at stages where the intestinal lumen has formed (Figure 8B). Second, contrary to our expectations, the percentage of upregulated genes at closer distances was higher than at 10Kb (Figure 8B). The density of upregulated adjacent genes varies with their distance from the Gypsy element, as shown in Figure 10C. Most of these upregulated genes are located closer to the Gypsy element, with gene density decreasing as the distance from the Gypsy element increases. This suggest that there is a correlation between gene expression and the distance of the genes to the Gypsy elements. This could be interpreted from two different perspectives: (1) the Gypsy elements upregulation could be a result of the transcription of their nearby genes, or (2) the upregulation of these genes is driven by the nearby Gypsy elements. However, further analyses would be required to address this.

### Extended gene families in the genome of the sea cucumber *H. glaberrima*

As has been shown so far, the genome of *H. glaberrima* provides exceptional opportunities to investigate and answer a diverse set of challenging questions that were not possible with transcriptomic information. Certain gene families, and particularly those that are extended in specific animal groups have been associated with the specific characteristics of the group. Thus, in the first sea cucumber genome to be sequenced, the authors associated the possibility that the observed extension of fibrinogen-related protein (FREP) and PSP94-like gene families could be associated to the regeneration prowess of holothurians [24]. In a similar way, we have identified distinct genes families that appear to be extended in the genome of holothurians. Studies of these gene families were initially limited to the characterization of individual transcripts and the extent of their importance or expansion within the genome was unknown. Now having a high-quality contiguous genome, we have the capability to describe them in more detail and assess their probable functional roles.

#### THAP Transcription Factor Family Extended in Species of Diverse Taxa

**Figure 9.**
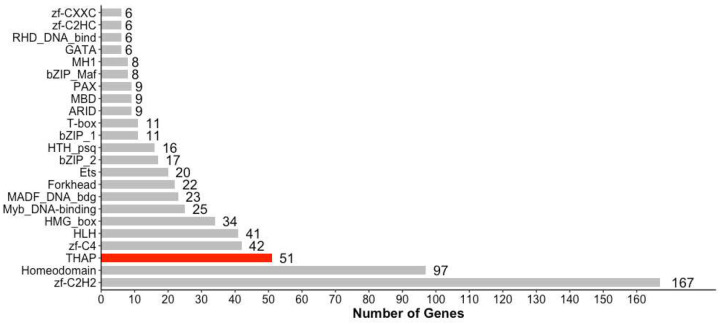
The sea cucumber *H. glaberrima* has a high number of THAP domain-containing genes. Bar plot of the total number of unique protein models of the largest 20 transcription factor families of the sea cucumber. Protein models were categorized with CREPE using HMMI profiles of known transcription factor families.

Among the gene groups that might be important candidates to the uniqueness of the regenerative capacities of the sea cucumber, are those molecules that promote biological processes that allow regeneration to occur. Among these are transcription factors, which are known to be important molecules for regulating complex developmental processes [63]. In addition, studies of transcription factors in regenerative species, such as the zebrafish and axolotl, have been shown to control the dynamics of their regeneration processes, such as cellular states transitions [64–66]. Yet, there is a lack of information about the transcription factors present in sea cucumbers and on how these compare to the known catalogue of transcription factors from other organisms. Getting deeper understanding about this transcription factors could certainly provide valuable insights into the differences of advanced regenerative species and those with limited regeneration capabilities. Here we have employed CREPE a tool designed to identify transcription factors using HMMI profiles that allow the identification and classification of the major known transcription factors domain families. This tool was able to catalogue gene peptide sequences for 51 different transcription factor families, with the zinc finger C2H2 (zf-C2H2) and homeodomain families being the largest with 167 and 97 genes, respectively (Figure 9).

**Figure 10.**
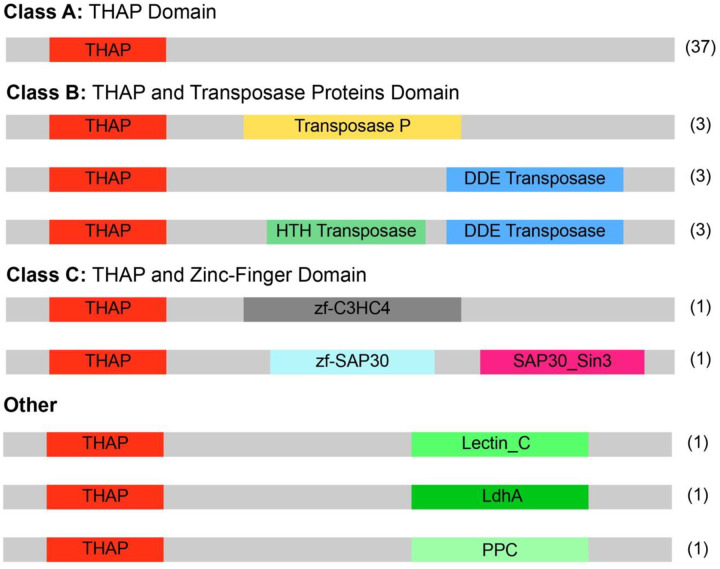
THAP domain-containing genes have additional variable domains that allows classification within classes. Schematic of the different domain combinations present in the THAP domain-containing genes of the sea cucumber *H. glaberrima* classified in different classes. The number in parenthesis to the right of each sequence schematic represents that total number of genes with that domain architecture. Lengths and location of the schematics do not represent the reality of the amino acid sequence structure. The order of the domains represents their order within the peptide sequence.

**Figure 11.**
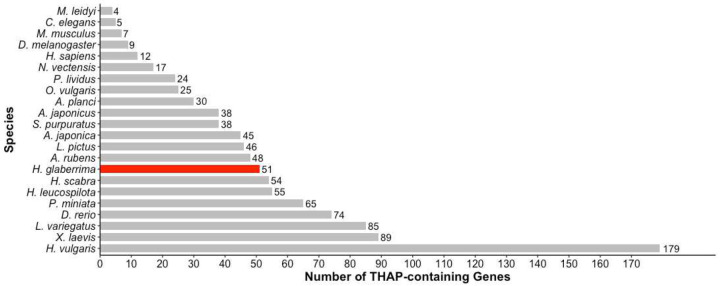
The number of THAP domain-containing genes varies greatly across taxa. Bar plot of the total number of unique protein models within the THAP domain transcription factor family identified with CREPE.

Interestingly, among the top 20 families identified the THAP (Thanatos-associated protein) transcription factors was the third largest family containing 51 protein models (Figure 9–10). This large number of THAP genes within *H. glaberrima* was surprising, as it is not among the traditional large families of transcription factors of animals, as is the case of the top two families identified. THAP genes have been found to be restricted to animal lineages. In humans, 12 THAP genes have been reported to be present and have been implicated in a diverse array of biological processes from transcription regulation, proliferation, pluripotency, cell cycle inhibition, and transposition [67]. The number of THAP genes present in humans was further confirmed with CREPE where 12 THAP domain containing genes were identified (Figure 11). Thus, our results reassure the number of THAP-containing genes in the human genome and further validate the capacity of transcription factor cataloguing of CREPE, providing confidence in the results obtained for the sea cucumber.

### Classification of the THAP transcription factor in *H. glaberrima*

Through further characterization of the 51 *H. glaberrima* THAP domain containing-genes, 3 distinct classes were identified to contain variable domains. To better describe this family, we have categorized these classes from THAP-A through THAP-C (Figure 10). Most of the THAP domain-containing genes had a single THAP domain (37 out of 51), which we refer to as THAP-A subfamily (Figure 10). In general, all peptide sequences were found to have a leading N-terminal THAP domain, except for one that had the THAP domain at the C-terminal end. In addition, THAP-B genes were characterized as containing transposase protein domains, where we find three groups with different domain composition. The first group of genes appear to be orthologs of THAP9, that are characterized by a transposase P domain (Tnp_P_element) (Figure 10). Two of these genes have comparable peptide sequence lengths to the THAP9 of humans having 819 and 828 amino acids, respectively, while the third gene codes for a 409-amino-acid sequence that could potentially be partial or a sequence that has been truncated due to its transposition activity. The second group of genes within the THAP-B class contain a DDE transposase endonuclease domain (DDE_Tnp_4) translating a peptide sequence with wide range of length from 233 to 425 amino acids (Figure 10). Lastly, the THAP-B class are characterized by the presence of DDE_Tnp_4 and a helix-turn-helix domain of DDE superfamily endonucleases domain (HTH_Tnp_4) (Figure 10). All genes within these groups showed the DDE_Tnp_4 domain at the C-terminal end after the HTH_Tnp_4 domain and codify a peptide sequence with a range of length from 413 to 569 amino acids (Figure 10).

The THAP-C class and the rest of THAP domain containing-genes are particularly interesting as they appeared to have developed specific features of molecule binding specificity. For instance, the THAP-C class is characterized by distinct zinc-finger domains, one has a region related to the RING U-box domain superfamily identified as the zf-C3HC4_2 domain, while the other had a SAP30 (SIN3 associated protein 30) zinc-finger domain (zf-SAP30) and an additional SAP30 conserved domain region, which is reported to be the binding region of SIN3 complex (Figure 10). The remaining THAP domain containing-genes that show features related to specific biological processes contained a conserved LdhA (lactate dehydrogenase) and Lectin_C (lectin C-type) domain, respectively (Figure 10). There was an additional THAP protein model that had a PCC domain, reported as being a conserved domain in plants and prokaryotes, in which region there was a putative zinc binding site (Figure 10).

### Echinoderms exhibit a large number of THAP genes

Other studies have shown that distinct taxa contain multiple THAP orthologs [68]. For instance, characterization of THAPs have shown a range from 6 THAP in chickens up to 32 in zebrafish [69]. Moreover, another recent publication on TE-related molecules also shows the expansion of THAP genes in specific animal species including cnidarian *Hydra*, annelid *Capitella*, and mollusk *Crassostrea* [70]. Thus, to assess the extended nature of the THAP genes in *H. glaberrima* we further characterized the number of THAP genes present in multiple echinoderms and animals from other phyla. For this we included the following species: echinoderms – sea cucumbers (*H. scabra, H. leucospilota, A. japonicus*), sea urchins (*S. purpuratus, P. lividus, Anchaster planci, Lytechinus pictus, Lytechinus variegatus*), the feather star (*Anneissia japonica*), and the sea stars (*Asterias rubens, Patiria miniata*), mollusk (*Octopus vulgaris*), mammals (*M. musculus and H. sapiens)*, fish (*D*. rerio), amphibian (*X. laevis*), euarthropod (*D. melanogaster*), nematode (*Caenorhabditis elegans*), cnidarians (*Nematostella vectensis* and *Hydra vulgaris*), and ctenophore (*Mnemiopsis leidyi*). For each species, we verified THAP protein models and removed isoforms to retain a single representative per gene. The transcription factor catalog of other echinoderms resulted in an extended number of the THAP transcription factor family genes ranging from 24 in *P. lividus* to 85 in *L. variegatus* (Figure 11). The large number of THAP genes in *L. variegatus* was surprising, being the sea star *P. miniata* the next echinoderm species with the greatest number of THAP domain-containing sequences with 58. Additionally, the number of THAP domain-containing genes identified in other holothuroids was comparable to the number of THAP genes of *H. glaberrima* with 55 in *H. leucospilota* and 54 in *H. scabra*, which further highlights the level of conservation of this transcription factor family within these taxa (Figure 11). Many of the THAP from this holothuroid species shared the same domain architecture with *H. glaberrima* THAP from all classes (THAP-A to - C). Nevertheless, in *H. scabra* no genes were found to have THAP/DDE or THAP/Tnp_P_4 sequences, while all shared the THAP/HTH/DDE domain architecture. The additional domains from the ‘other’ category of *H. glaberrima* were not found in any of the two holothuroids except for THAP/PPC. Both holothuroids also showed presence of additional domains that were unique to each. Furthermore, the sea cucumber *A. japonicus* has a lower number of THAPs with 38 total sequences, which potentially is correlated with its genome length as it is almost half the genome size of the holothuroid species (Figure 11). While we understand the number of identifiable THAP genes is dependable on the quality of the protein models being analyzed the number of genes identified within the holothuroid sea cucumber group provides sufficient confidence that the number of genes identified across species is correct. In addition to THAPs in humans, much of the results from our THAP catalog of other species is aligned with previous reports including the number of THAPs found on *O. vulgaris* (25), *D. melanogaster* (9), *M. leidyi* (4), *M. musculus* (7), and *H. sapiens* (12) [69, 70]. In contrast, the number of THAPs we identified of other species was different from those previously reported after removal of isoforms as for *N. vectensis* (17 vs 15), *H. vulgaris* (73 vs 179), *C. elegans* (5 vs 8), *D. rerio* (65 vs 32), and *X. laevis* (89 vs 23) [69, 70]. Notably, the approach of THAP identification and data available through the years for each analysis quite different. From the species that we assessed only *Hydra* showed a dramatic number of THAP sequences, even after removal of isoforms. The rest of the species including ctenophores, mollusks, arthropod, nematode and chordates (frog, mouse, chicken, human) showed lower number of THAP genes from 4 in *Mnemiopsis* to 25 in the Octopus (Figure 11). The diverse number of THAPs across distinct taxa is quite drastic but reflects the evolution across species and potentially the structural changes of their genomes.

#### Scavenger receptor Cysteine-Rich (SRCR) Containing Gene Family

In many of our previous transcriptomic experiments aimed at characterizing genes associated with regenerative processes, we frequently identified sequences containing Scavenger Receptor Cysteine-Rich (SRCR) domains. A substantial number of these sequences were annotated as *Neurotrypsin*, *Deleted in Malignant Brain Tumor 1* (DMBT1), or other SRCR genes. With the genome in hand, we assessed the similarities, differences, and genomic organization of *H. glaberrima* SRCR genes.

To this end, we analyzed transcriptomic datasets derived from the intestine and radial nerve cord of *H. glaberrima*. Candidate SRCR transcripts were identified through BLAST searches using orthologs of *Neurotrypsin*, *DMBT1*, and representative SRCR genes as query sequences. These searches yielded a total of 58 transcripts from the radial nerve cord transcriptome and 56 transcripts from the intestine transcriptome.

Open reading frame (ORF) analyses were subsequently performed on all 114 transcripts. Transcripts exhibiting high sequence redundancy were excluded, as were those containing incomplete ORFs. Following this filtering process, 34 complete transcripts were retained, half derived from the intestine transcriptome and *half* from the radial nerve cord transcriptome. These transcripts were then aligned against the *H. glaberrima* genome using BLAST to identify their corresponding genomic sequence and loci. In total, we identified 37 SRCR-containing genomic loci coding for putative proteins (Table S3).

SRCR domains have been classified according to the number and position of conserved cysteine residues [71, 72]. This classification groups SRCR domains with 6 Cys in group A and those with 8 Cys in group B. According to this classification most if not all *H. glaberrima* SRCR domains described here belong to group A.

We then extended the analysis of our sequences to examine the size of the putative encoded proteins, the number of SRCR domains, the presence of additional domains, and their predicted scaffold localization (Table S3). Our results revealed a wide diversity of proteins, ranging in size from fewer than 200 amino acids to approximately 5,981 amino acids—nearly thirty times larger. Similarly, the number of SRCR domains varied substantially, from 1 to 51. In addition, these proteins contain a large variety of other domains. However, the diversity and variability of these domains were so extensive that they did not provide a useful basis for further classification.

**Figure 12.**
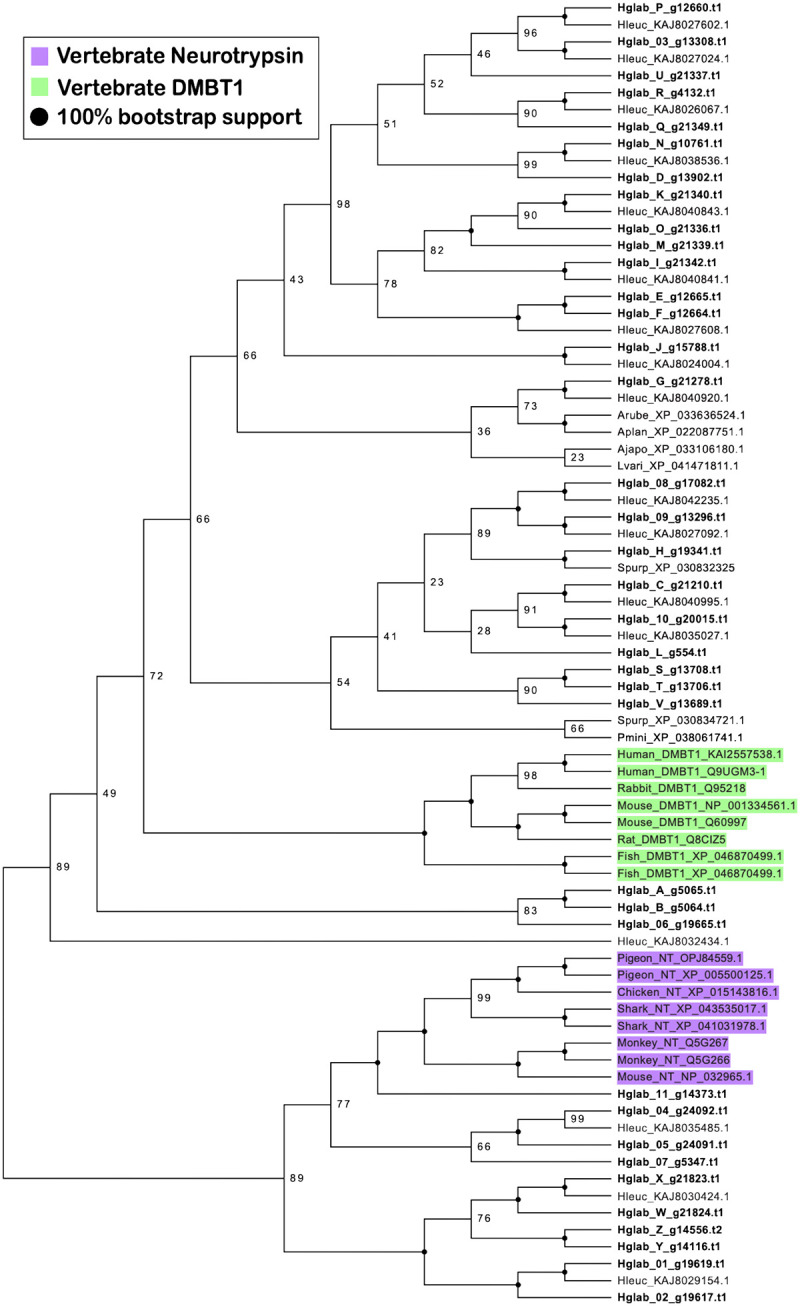
Maximum-likelihood phylogenetic tree of vertebrate and echinoderm SCRC gene sequences. Vertebrate DMBT sequences (green) form a well-supported clade that is sister to branches containing echinoderm sequences, including H. glaberrima (bold). Similarly, vertebrate neurotrypsin genes (purple) cluster into a distinct clade positioned adjacent to sequences from *H. glaberrima* (bold) and *H. leucospilota*. Black dots indicate nodes with 100% bootstrap support.

The resulting phylogenetic tree separated the sequences into two major clades: one containing DMBT proteins and the other containing neurotrypsins (Figure 12). Twenty-four *H. glaberrima* sequences clustered with vertebrate DMBTs and those from other echinoderm classes, including sea cucumbers (*H. leucospilota* and *Apostichopus japonicus*), sea stars (*Asterias rubens* and *A. planci*), and sea urchins (*Strongylocentrotus purpuratus* and *Lytechinus variegatus*). A second clade included ten *H. glaberrima* sequences that grouped with vertebrate Neurotrypsins. This second clade included *H. leucospilota* sequences, but no sequences from non-holothuroid echinoderms. However, this might be due to an incomplete selection of SRCR sequences, and an extended study is still needed to determine if other echinoderm genomes have neurotrypsin genes. Three *H. glaberrima* sequences and one *H. leucospilota* sequence had SRCR domains but did not cluster cleanly with either vertebrate DMBT or neurotrypsin clades.

We further analyzed the size of the putative proteins and the number of SRCR domains within both clades. The average size of the putative *H. glaberrima* proteins in the DMBT clade was approximately 1.4 times that of the *H. glaberrima* proteins in the Neurotrypsin clade. Likewise, the average number of SRCR domains was higher in the *H. glaberrima* sequences of the DMBT clade (5.3) compared with those in the Neurotrypsin clade (2). The three sequences that cluster separately show wide ranges in size and number of SRCR domains.

## Discussion

### Sea Cucumber Genome Assemblies Demonstrate High Conservation

In the present work we have assembled the most complete genome of the sea cucumber *H. glaberrima* to date with an N50 of 50.8 Mb, L50 of 11 and 94% of genomic data in the largest 23 scaffolds (Table 1). In addition, we have now reduced the gene predictions by 35% to that of our previous publication [16] (53,080 to 34,720) increasing our BUSCO complete score by more than 10% (73.06% to 85.64%). This genome compares favorably with other echinoderm assemblies in that it achieves comparable contiguity (e.g., *S. purpuratus, A. planci*), while providing a high percentage of annotated protein-coding genes and a thorough representation of repetitive elements. Moreover, our analyses and results are comparable to genome assemblies that have been referred to by authors as chromosome level assemblies, based on genetic linkage-maps or, as in this study, chromatin conformation capture sequencing linkages (i.e., 2b-RAD or Hi-C) [19, 23, 24]. In recent years, the increasing interest in sea cucumbers has provided a wider amount of genomic information to become available. Yet, genomic information for sea cucumber species that are used as model systems in experimental research remains limited. Until now, only one species that is used for regeneration studies, the sea cucumber *A. japonicus*, had a publicly available genome assembly and annotation [24]. However, the high heterozygosity shown to be present within sea cucumber species makes it challenging to utilize this information as a reference for molecular studies in other species, such as the model species *H. glaberrima*.

Our assertion of having a high-grade genome assembly is further sustained by the analyses of two well-known gene families, Hox and Sox. Consistent with other animals, we show a high level of conservation in presence/absence and genomic and genomic distribution in these genes (Figure 3). For instance, we find that the Hox genes are organized in a conserved cluster in the same order within a single chromosome (i.e. chr10), shown to be the analogous chromosome to *H. leucospilota* and *A. japonicus*, chromosome 9 and 10, respectively (Figure 2A). The structural genomic conservation of Hox genes highlights the probable organizational specificity required for the roles that these genes have, as has been shown for other animals previously [47, 48, 51, 52, 73]. In addition, the homologous *Hox* organization between sea cucumbers and the hemichordate *S. kowalevskii*, and the differences to the sea urchin *S. purpuratus*, alludes to the importance of *Hox* in determining the body patterning of these organisms.

We also demonstrate genomic organizational conservation of the Sox gene family and display the known cross-phylum conservation. The latter was shown by our phylogenetic analysis where all the *M. musculus* Sox members from individual classes, clustered with the corresponding echinoderm representative for each class. The organizational conservation of Sox genes in the sea cucumbers is also shown in other vertebrate species. For example, in the buffalo and cow the members of the class B1 and B2, Sox2 and Sox14, are in chromosome 1 [74], whereas the orthologous genes, SoxB1 and SoxB2, are also clustered in the same syntenic chromosomes in the sea cucumbers *H. glaberrima, A. japonicus*, and *H. leucospilota* (Figure 4B). These characterizations and macrosynteny analyses show that sea cucumbers share much of their genomic architecture, while exhibiting some differences at the genus level (Figure 2). Nevertheless, we find fascinating how sequencing technologies and assembly pipelines have allowed the generation of genomic information that is comparable between species of the same genus without the need of extensive references or additional experimental techniques. Through this, we have shown *H. glaberrima* to have comparable genomic characteristics to the genetic organization of the latest reported genomes of other holothuroid sea cucumbers, including *H. leucospilota* [19] and *H. scabra* [20]. Thus, we ascertain that our newly assembled genome of *H. glaberrima* is comparable to other high-quality echinoderm genomes as to be useful for evolutionary, genomic, developmental, and functional studies, providing a robust framework to decipher the genetic basis of the regenerative capacities of holothurians.

### Comparative representation of transposable elements: echinoderms and vertebrates

Having established the quality of our newly assembled genome, we can now use it to address questions related to the unique characteristics of holothurians and echinoderms more broadly. Among the many conundrums that encircle sea cucumbers is the possible role of TEs. These are still among the least studied aspects of genomes despite being shown to be important genomic features that cover around 50% of all animal genomes [75]. Increasing evidence suggests that these elements are important regulators of gene expression and that they are linked to multiple human diseases [76, 77]. Similarly, the major classes of TEs have been shown to have differential genomic coverage in different species, a characteristic that has been suggested to be correlated with the evolution of species from diverse phylum [75, 78, 79]. Retrotransposons in particular have been linked to regeneration and pluripotency, acting as binding sites of pluripotency transcription factors in pluripotent stem cells (PSCs) [80], and comprising 59% of the highly regenerative axolotl genome [58]. In contrast, the 50% of the genome of *H. glaberrima* covered by TEs, only ~ 8% correspond to LTR elements, being the second largest group after DNA transposons (Table 3 and Figure 5). Interestingly, the distribution of these TEs, including Gypsy retrotransposons, does not show an insertion preference, in contrast to LINE-1 elements in mammals [81].

Nowadays, in many genome assembly pipelines, repetitive regions (including TEs) are identified and masked using RepeatMasker, a program that performs repetitive sequence identification, but does not provide a robust annotation of TEs, particularly retrotransposons. However, if we look at previous publications where the TE content is documented, the overall genomic coverage of TEs identified using EDTA agrees with our findings. For instance, in *H. leucospilota* we find a repetitive sequence genome coverage of 50.31% congruent with the 50.41% reported with its genome assembly [19]. Similarly, for *A. japonicus* our analysis resulted in 29.41% repetitive sequence genome coverage which is close to the 26.20% reported [56]. A final example is that of *Drosophila* for which we found ~ 18% genome coverage of repetitive sequences, close to the 20% previously reported [82].

Furthermore, for this same species our findings agree at the subfamily level as we have identified over 11% LTR genome coverage which is once again congruent with the 12% reported [82]. Having validated this pipeline across diverse species has allowed us to perform robust comparative analyses of TE composition. It is important to note that the ability to adequately annotate identified TEs is dependent on the quality of the assembled repetitive genome, which for many years has been technically challenging, and is further complicated by the structural corruption of individual elements caused by new TE insertions.

Although expanded TE abundance has been proposed to correlate with regenerative capacity, comparing TE composition across species with varying regenerative capacities, including the sea cucumber *H. glaberrima*, *H. leucospilota* (~50%) [19], and *A. japonicus* (~47%) [22], the moderately regenerative zebrafish *D. rerio* (~52%) [83], and the less regenerative *S. purpuratus* (~39%) [84], *X. laevis* (~65%) [85], *M. musculus* (~45%) [86], and *D. melanogaster* (18%) [82] reveals no clear relationship between TE percentage and regenerative ability. Notably, *S. pupuratus*, shows LTR coverage comparable to that of the holothuroids (data not shown), and *D. melanogaster*, arguably the least regeneratively competent species in the comparison, shows LTR coverage closer to that of echinoderms [82] and zebrafish than to mammals. What emerges is not a regeneration-capacity gradient but rather a phylogenetic signal: mammals consistently show lower LTR representation than non-mammalian species, a pattern better explained by lineage-specific TE dynamics than by regenerative capacity [87, 88]. Therefore, all this together suggests that gross TE genomic content, including LTR element abundance, is not a reliable predictor of regenerative capability.

While there seems to be no support of a correlation between retrotransposon abundance and regeneration potential, the question behind the elevated expression of these elements during regeneration remains. It may be more informative to consider their transcriptional activity rather than their genomic abundance. Several studies have documented distinct retrotransposons to be highly upregulated during regeneration. Among the first reports was a study by our group describing *Gypsy* and BEL LTR families differentially expressed at different stages of radial nerve regeneration in *H. glaberrima* [60]. In the same species, it was later reported that two Gypsy retrotransposons were also expressed at various stages of intestinal regeneration [61]. In the axolotl the non-LTR LINE-1 was shown to be activated during limb regeneration [89], while the Iberian ribbed newt, *Pleurodeles walt*, also showed that specific TE, including Gypsy elements, were upregulated during limb regeneration [62]. A final example was a study on lungfish tail regeneration where the authors found 16 TE transcripts upregulated in the regenerating tail blastema [90]. In the present study, we confirmed the overexpression of *Gypsy1* and *Gypsy2* and demonstrated the expression of additional Gypsy elements across different stages of intestinal regeneration in *H. glaberrima*. This accumulated evidence from sea cucumbers and other species provides a mounting support for a functional link between specific retrotransposon groups and regeneration, independent of their overall genomic abundance.

One argument raised to explain TE upregulation during regeneration is that it is a transcriptional byproduct of nearby gene activation rather than a driver of it. The correlation we observed between overexpressed Gypsy elements and elevated transcription neighboring genes could be explained with two distinct hypotheses: (1) the Gypsy elements are upregulated during regeneration as a consequence of the transcriptional activity of nearby regeneration-associated genes or (2) the Gypsy elements themselves serve to promote the transcription of the nearby genes that need to be expressed during regeneration. Interestingly, *Gypsy1* showed its highest level of upregulation at 12HPE, yet the highest percentage of upregulated nearby genes was observed at 14DPE (only in the anterior and posterior regions) and 21 DPE (Figure 8), timepoints at which *Gypsy1* and most other Gypsy elements remain highly expressed. This temporal dissociation may suggest that the relationship is not purely a byproduct effect.

We find it notable that the percentage of genes near the Gypsy elements that are upregulated is higher at stages of regeneration where the lumen of the intestine has formed. A potential explanation for this, is that at these advanced stages there are more tissue layers within the regenerating organ and thus the number of genes expressed increases. However, considering that there is also a high expression of *Gypsy* elements at late stages of regeneration, we could infer that these are in some way influencing the expression of other genes potentially reflecting the increased transcriptional complexity associated with a more differentiated, multi-layered organ. Nevertheless, given the sustained high expression of Gypsy elements at these late stages, a regulatory role cannot be dismissed. LTR elements could act as transcriptional regulatory regions [91], and prior studies in *Drosophila* have demonstrated that Gypsy elements can act as insulators capable of regionalizing chromatin domains to promote or inhibit gene expression [92–94]. More recent studies in mouse have shown that retrotransposons may act as promoters of transcription of specific genes [95, 96]. Taken together, these findings suggest that while overall TE abundance does not predict regenerative capacity, the context-dependent transcriptional activity of specific TE families, particularly Gypsy elements, may play a meaningful regulatory role during regeneration. The involvement of these elements in regeneration may therefore transcend simple questions of copy number or genomic coverage.

### New extended gene families in holothurians

As shown, the availability of high-quality genomes of echinoderms, and in particular holothuroids, has provided us with amazing opportunities to correlate their unique features at the physiological level to their genomic characteristics. Among echinoderms, there are numerous characteristics that make sea cucumbers stand out within this phylum, such as their adult bilateral symmetry, distinct bodily structures, unique defense mechanisms, and robust regeneration capacity. Because of these fascinating traits, sea cucumber genomics goes back many years.

The first sea cucumber genome assemblies, that of *Parastichopus parvimensis*, and *Australostichopus mollis* [97] were highly fragmented and offered only glimpses of the complexity of their genomic structure and components. Nevertheless, more recent genome assemblies, including the current study, have led to a richer understanding of the genomic architectures underlying these unique species. One of the most interesting findings is the extended and species-specific gene families found in the reported sea cucumber genomes. For example, the first high-quality sea cucumber genome reported from *A. japonicus*, showed that five specific gene families appeared to be expanded when compared to other animals: fibrinogen-related proteins (FREPS), retrovirus-related Pol polyprotein from transposons, nucleotide-binding oligomerization domain-like receptor (NLR) family caspase recruitment domain (CARD) domain-containing proteins, tyrosine-protein kinase receptors, and zing finger CysCysHisCys (CCHC) domain-containing proteins [24]. Similarly, a recent publication on the genome of the holothuroid *Chiridota heheva* showed that 66 gene families related to cell cycle progression, protein folding, and ribosome assembly, appeared to be expanded in this species compared to non-holothuroid echinoderms [98]. The recent genome of the sea cucumber *Paelopatides* sp., which comes from the deep-sea environment of the Yap trench, strikingly shows a total of 6,030 expanded gene families when compared to *S. purpuratus* and *A. japonicus*, with 173 expanded functional domains associated with transposons, genetic information processing, immunity, material transport, apoptosis, metabolism, and DNA binding [99]. We believe that this number of expanded gene families is too high and should be taken with precaution as it is highly probable that this large number of expanded gene families reflects the quality of the genome assembly, as DNA from three sea cucumbers was utilized, the specimens was not in optimal conditions and the final genome assembly contains a large number of scaffolds (55,447). Adding to the repertoire of expanded genes in distinct sea cucumber species, the draft genome of the sea cucumber *Stichopus monotuberculatus* showed specific gene expansions that are suggested to be key to their characteristics [25]. Among the 857 gene family expansions that resulted from comparisons with other echinoderms there were gene families related to key enzymes involved in fucosylated chondroitin sulfates (FCS) biosynthesis, such as carbohydrate sulfotransferases (CHST) and fucosyltransferases (FUT). Nonetheless, it is important to take into consideration that from all the expanded gene families only a handful have been confirmed and studied in detail and could potentially be a representation of artefactual information within the corresponding assemblies. However, in the case of *S. monotuberculatus*, this number of expanded genes families is closer to expansion seen in other species. For example, a study in mammalian lineages representing 93 million years the mouse showed the highest expansion with 714 expanded gene families [100]. In other studies, focused on domesticated cattle, and where humans where included, the largest expansion observed was of 739 gene families [101].

The expanded gene families that have been studied in detail in many cases have been suggested to correlate to specific characteristics of the sea cucumbers. For example, the expansions of the FREPS and prostatic secretory protein of 94 amino acids-like (PSP92-like) gene families in *A. japonicus* have been suggested to correlate to its regenerative capabilities [24]. Similarly, in an *A. japonicus* genome assembly performed by another group [23], the expansion of the fibroblast growth factor receptor (*Fgfr*) gene family was also reported and proposed to be correlated with the sea cucumber regeneration. In a similar way, our laboratory has also reported expansion of candidate gene families such as *Melanotransferins*, which are highly expressed during regeneration [16, 102]. Furthermore, a recent study in the sea cucumber *H. leucospilota* is perhaps the best example of the adequate approach to establish a relationship between the expansion and functional role of a gene family [19]. In the study the authors describe unique molecules with tandem repeat sequences and expansion of Ambulacraria-specific ligand-gated ion channel (LGIC) genes (nAchR-like LGIC genes) and demonstrate through *in vivo* and *in vitro* experiments their relationship to the characteristics of the Cuverian organ and their expulsion [19].

The presence of extended genomic features, nonetheless, is also found in the sea cucumber *H. glaberrima*, which we have shown for the LTR retrotransposons, THAP, and SRCR domain-containing genes. In fact, we are careful not to assign a particular function without further examination and believe these gene feature might be the genomic underpinnings of unique biological processes of *H. glaberrima* and other sea cucumber species, physiological or developmental functions, beyond their regenerative capacity. All of which demonstrates the importance of studying the genomic characteristics of these species to advance our knowledge of their activity and broader correlation within molecular networks that underly the amazing mechanisms that this animals exhibit.

#### New extended THAP transcription factor family in echinoderms

*H. glaberrima* appears to have three times more copies of THAP genes when compared to vertebrates. This group of genes has not been described in detail in echinoderms. Interestingly, THAP genes have been shown to be closely associated with TEs, where THAP9, for example, was reported to mobilize TEs and help in their domestication [103], extending our analyses of TEs in holothurians. Here for the first time, we have identified and characterized the full complement of THAP genes in a holothuroid species and broadened the identification to include various other species (Figure 11). THAP genes were first identified in humans with each of the 12 distinct genes (*THAP0*-*THAP11*) containing a single THAP conserved domain [68]. The THAP genes of humans appeared to form a very simple clade with only two of its members showing an additional domain, *THAP9* with a transposase P element domain and *THAP11* with a polyglutamine track [68]. Recently, a study on these THAP genes revealed a coiled-coil conserved region across all the THAPs despite their length, except for THAP10 [67]. The expanded THAP gene repertoire of *H. glaberrima* is consistent with what has been shown in other metazoans [70]. Of the 51 THAP domain-containing genes identified in *H. glaberrima*, 37 share a homologous structure with the single THAP domain-containing genes of humans (THAP0–10), constituting THAP class A (THAP-A) (Figure 10). There are studies from *Drosophila* and *C. elegans* reporting THAP with multiple THAP domain repeats within a single gene [69]. Such multiple THAP domain repeats within a single gene are absent in *H. glaberrima* and other holothurians, as well as in humans, mouse, and zebrafish, though they are present in other echinoderms including the sea star *P. miniata* and sea urchin *L. variegatus*. Genes with domain architectures similar to human THAP9, carrying the additional TE-related transposase P domain, along with other groups of genes containing TE-related domains, have been collectively categorized as THAP class B (THAP-B) (Figure 10). Notably, genes with homologous structure to the *H. glaberrima* THAP-B class have also been found in other species including the echinoderms assessed here and previously reported species such as the mollusks *Crassotrea gigas* and *Aplysia, Hydra*, and *Amphimedon* [70]. However, as mentioned earlier, humans only contain 1 copy, whereas the species mentioned above contain multiple copies of THAP/TE-related domains. A third class (THAP-C) encompasses genes containing additional zing finger-binding domains, a feature present in some echinoderms, *Aplysia, C. elegans* and *Drosophila*, but absent in humans (Figure 10).

Based on these results, and those of previous studies [70], it is suggested that the expansion of the distinct THAP genes appears to be independent in distinct animal species, which is primarily observed in their differences in domain architecture and copy number. For instance, a recent publication shows two cnidarians, *Nematostella* and *Hydra*, with a substantial difference in the copy number of THAP domain-containing genes (15 vs 73) [70]. Similarly, within echinoderms we find a great variety in the accessory domains associated with THAP genes. The presence of TE-related domains in sea cucumber THAP genes, shared with Hydra, *Crassostrea gigas*, and *Amphimedon* bur largely absent in vertebrates, further underscores the lineage-specific nature of THAP gene evolution [70]. Interestingly, there appears to be a THAP gene in *H. glaberrima* that has a lactate dehydrogenase domain (Figure 10), like one of the THAP domain-containing genes (*CTBP1*) in *C. elegans* that has been reported to act as a transcriptional co-repressor molecule [69, 104]. This domain is not present in any of the species analyzed so far nor in other sea cucumbers.

Another interesting characteristic of THAP domain-containing genes is that these genes are restricted to animals, with no reports of THAPs present in plants, fungi, yeast or bacteria [105]. In addition, these genes have since been correlated with specific biological processes, such as apoptosis, cell proliferation, chromatin compartmentalization, stem cell pluripotency, among other [69, 106]. Therefore, the numerous copies of THAP genes within the echinoderms and other animals could certainly reflect the importance of these genes in specific biological process. In fact, a THAP gene, with high similarity to THAP6, has been previously shown to be among the top expressed genes during the early stages of intestinal regeneration of the sea cucumber *H. glaberrima* [13]. Therefore, our analysis provides additional evidence that supports previous reports of the lineage-specific expansion and domestication of the THAP genes across animals, potentially due to their role as key molecules for distinct processes in these species.

#### Scavenger receptor Cysteine-Rich (SRCR) Containing Gene Family

The integration of genomic resources with transcriptomic analyses provides a powerful framework for accurately characterizing gene families with complex domain architectures. Transcriptome assemblies alone can be limited by incomplete transcripts, fragmented contigs, or misassembled sequences, particularly for genes containing repetitive domains such as SRCR motifs. By anchoring transcript sequences to the genome, we were able to verify exon–intron boundaries, confirm domain organization, and distinguish between closely related gene models. This genome-guided approach significantly improved the resolution of SRCR gene identification and reduced the likelihood of misclassification caused by partial or chimeric transcript sequences.

SRCR proteins belong to a large protein superfamily characterized by substantial diversity in sequence, tissue expression, and other biological features [107]. This structural variability likely reflects a broad range of functions; however, in general, SRCR proteins are most commonly associated with immune-related roles [108]. The SRCR gene family been shown to have a large heterogeneity at both genomic and transcriptional levels in invertebrates and in sea urchins and sponges it has been shown to be expanded [72, 109]. Our results show that this large heterogeneity is also found in holothurians.

Our analyses indicate that the SRCR proteins identified in the holothurian transcriptome shared some similarities to DMBT1s and/or Neurotrypsins. Moreover, phylogenetic analyses and comparisons of domain architecture further resolved these sequences into two major clades corresponding to DMBT1-like proteins and neurotrypsin-like proteins. DMBT1 proteins though associated with mucosal immunity and pattern recognition have also been found, as the name describes, to be associated with nervous system functions [110]. Neurotrypsins, first identified in the nervous system (thus their name) are secreted serine proteases that contain SRCR domains and participate in extracellular proteolytic signaling pathways [111]. The placement of the holothurian sequences within these clades indicates that both functional categories are conserved within echinoderms and suggests that these proteins likely mediate ligand recognition or protein–protein interactions within immune or extracellular regulatory processes.

Structural characteristics of the holothurian proteins further support their classification into these two families. Proteins clustering within the DMBT1 clade were on average approximately twice as large as those within the neurotrypsin clade and contained a higher number of SRCR domains. This observation is consistent with the canonical architecture of these protein families where, in vertebrates, DMBT1s have been reported as consisting of 2,000–3,000 amino acids and having around 13 SRCR domains, while neurotrypsins have around 700–900 amino acids and have less than 4 SRCR domains [110, 111].

An important observation emerging from our analyses is that a substantial proportion of SRCR proteins currently annotated in echinoderm genomic databases appear to be incorrectly classified. In several cases, sequences annotated as DMBT1 were found, based on phylogenetic placement and domain architecture, to cluster clearly with neurotrypsin-like proteins or viceversa, those annotated as neurotrypsin were found in the DMBT1 branch. The modular and repetitive nature of SRCR domains likely contributes to these annotation inconsistencies. Because SRCR motifs frequently occur in multiple copies and may coexist with other repetitive or low-complexity domains, automated annotation algorithms can incorrectly split or merge gene models, leading to inaccurate predictions of protein size and domain composition. In addition, incomplete transcript sequences derived from transcriptome assemblies can obscure the full domain architecture of these proteins, further complicating annotation in the absence of genomic information that allows verification of exon–intron organization.

The availability of the *H. glaberrima* genome allowed us to overcome many of these limitations by enabling genome-guided transcript validation and gene model reconstruction. By mapping transcript sequences to the genome, we were able to confirm complete coding sequences, identify corresponding genomic loci, and reconstruct the full domain architecture of the encoded proteins. This approach not only improved the accuracy of SRCR gene identification but also clarified the evolutionary relationships of these sequences within the SRCR superfamily.

## Conclusion

In conclusion, our work presents a new and improved genome assembly for *Holothuria glaberrima*. This resource adds an additional high-quality genome to the class Holothuroidea, a group for which genomic resources have expanded considerably in recent years but remain relatively limited compared with other echinoderm classes. The availability of this genome therefore contributes to a growing comparative framework that will facilitate evolutionary, genomic, and functional studies across echinoderms.

Beyond the assembly itself, our analyses provide new insights into several genomic features that have not previously been explored in detail in holothurians. These include an in-depth characterization of the transposable element component of the genome, as well as new information on the organization and diversity of several gene families. Such analyses provide a broader understanding of genome structure and evolution within Holothuroidea and create a foundation for future comparative genomic studies aimed at understanding lineage-specific innovations and regulatory complexity.

Nevertheless, the most significant contribution of this genome is likely to be its impact on ongoing and future studies that use *H. glaberrima* as an emerging model system for regeneration biology [112]. This species has long been recognized as a powerful experimental organism for investigating regenerative processes, particularly the remarkable capacity to regenerate both intestinal tissues and components of the nervous system. The availability of a well-annotated genome greatly enhances the ability to identify genes, reconstruct signaling pathways, and analyze gene regulatory networks involved in these processes.

Indeed, the impact of this genomic resource is already evident. Two recent studies have directly benefited from the availability of the *H. glaberrima* genome. One study employed single-cell RNA sequencing to investigate the cellular dynamics underlying intestinal regeneration [113], while another focused on identifying ligand–receptor interactions involved in the re-innervation of the regenerating intestine [114]. In both cases, the genome provided the essential framework required for accurate transcript annotation, gene identification, and downstream functional interpretation. Looking forward, the availability of this genome will greatly expand the range of experimental and computational approaches that can be applied to *H. glaberrima*. These include high-resolution transcriptomics, epigenomic analyses, gene regulatory network reconstruction, and functional genomic studies aimed at identifying the molecular mechanisms that underlie regenerative capacity. Given the increasing interest in understanding regeneration from an evolutionary and biomedical perspective, *H. glaberrima* is poised to become an increasingly important model organism in the field. For these reasons, it is reasonable to expect that the availability of this genome will substantially accelerate research using *H. glaberrima* and significantly increase the number and scope of studies addressing regeneration, developmental biology, and genome evolution in this species.

## Supplementary Material

1

This is a list of supplementary files associated with this preprint. Click to download.

- TableS3.xlsx

## Figures and Tables

**Table 1.** General Genome Assembly Metrics and BUSCO Completeness Assessment Results.

General Metric		
	No. of Sequences	2,619
	Total Length	1,234,837,113
	N50	50,898,579
	N90	34,873,900
	L50	11
	L90	22
	Maximum Scaffold Length	73,668,981
	No. of scaffold > 1kb	2,612 (99.7% of total)
BUSCO Statistics (N = 954)		
	Complete	844 (88.47%)
	Complete + partial	885 (92.77%)
	Complete and single copy	231 (90.59%)
	Complete and duplicated	1.6%
	Fragmented	4.3%
	Missing	69 (7.23%)

**Table 2.** Gene model predictions statistics for distinct pipelines.

Metric	AUGUSTUS	BRAKER3	TSEBRA	EVM
Genes	34,720	20,121	42,217	30,363
Longest sequence (aa)	79360	4,555	9,360	9264
Shortest sequence	7	3	3	50
Mean sequence length	414	401	403	373
Median sequence length	294	320	290	267
**BUSCO Assessment (*n*=954)**				
Complete	817 (85.64%)	685 (71.80%)	822 (86.16%)	805 (84.38%)
Complete + partial	883 (92.56%)	765 (80.19%)	888 (93.08%)	879 (92.14%)
Complete and single copy	81.3%	62.9%	69.3%	83.2%
Complete and duplicated	4.3%	8.9%	16.9%	1.2%
Fragmented	6.9%	8.4%	6.9%	7.8%
Missing	7.5%	19.8%	6.9%	7.8%

**Table 3.** Results of the annotation of transposable elements on the genome of *H. glaberrima* with different tools.

	Repeat Masker			EDTA v2.1			EDTA v2.2		
Class	Count	Total bp	Gen. %	Count	Total bp	Gen. %	Count	Total bp	Gen. %
**Retrotransposons**	**186212**	**80904327**	**6.54%**	**176991**	**78593063**	**6.36%**	**340371**	**14363439**	**13.96%**
LINE	129863	65134333	5.26%	12027	6602152	0.53%	103798	51702184	4.20%
SINE	41260	6392306	0.52%	-	-	-	72518	15476906	1.25%
LTR:	129863	65134333	0.76%	164964	71990911	5.83%	238090	92635030	7.50%
BEL/Pao	629	693163	0.06%	-	-	-	1517	702817	0.06%
Copia	234	67716	0.01%	2117	676137	0.05%	982	351270	0.03%
**Gypsy**	**13220**	**8086626**	**0.65%**	**43329**	**26753604**	**2.17%**	**22865**	**15466326**	**1.25%**
Unknown	-	-	-	119518	44561170	3.61%	212726	76114617	6.16%
DNA Transposons	92038	24723070	2%	1501772	494903950	40.07%	1297888	38484756	31.17%
Unclassified	2162714	553090009	44.65%	-	-	-	-	-	-
**Total interspersed repeats**		**658863189**	**53.18%**		**628480694**	**50.90%**		**58685228**	**47.53%**

## Data Availability

The genome assembly and annotation generated in this study has been made available in FigShare (doi: 10.6084/m9.figshare.31743223). Associated code has been made available in GitHub at https://github.com/joshuagmedina/hglaberrima_ref_genome.git.
