# Supplementary material for: Sea Cucumber Holothuria glaberrima High-Quality Genome: new and extended gene families in Holothuroidea
Source: Res Sq. 2026 May 20:rs.3.rs-9490613. Preprint. [Version 1] doi: 10.21203/rs.3.rs-9490613/v1 (PMC13228809; doi:10.21203/rs.3.rs-9490613/v1)
Supplement: 1 [file NIHPPrs9490613V1-supplement-1.pdf]

Supplemental Data

Table S1. General metrics of the genome assembly of various sea cucumber species.

Species	Size (Gb)	Scaffolds	N50 (Mb)	N90	Genes	Ref
H. glaberrima	1.23	2,619	50.9	34.9 Mb	34,720	-
H. glaberrima Draft	1.10	89,105	0.025	-	53,080	Medina-Feliciano et al. 2021
H. leucospilota	1.39	2,312	56.10	42.3 Mb	36,089	Chen et al. 2023
H. scabra	1.19	31	53.52	39.5 Mb	34,418	Zhong et al. 2024
A. japonicus 2023	0.67	34	29.65	-	19,828	Sun et al. 2023
A. japonicus 2018	0.95	7,286	0.196	-	29,451	Li et al. 2018
A. japonicus 2017	0.81	3,281	0.486	-	30,350	Zhang et al. 2017
S. monotuberculatus	0.84	168	11.51	-	36,422	Zhong et al. 2023
Chiridota heheva	1.11	4,609	1.22	-	36,527	Zhang etl a. 2022

**Table S2. OrthoFinder results per each of the species included in the analysis.**											
	**Ajap2017**	**Ajap2023**	**Amex**	**Drer**	**Hglab**	**Hleu**	**Hsap**	**Hsca**	**Mmus**	**Pliv**	**Spur**
Number of genes	30221	19828	112408	52089	34720	33857	69839	34418	84985	50803	38439
Number of genes in orthogroups	29021	18244	105472	50171	30412	30811	68821	28397	83539	41629	37347
Number of unassigned genes	1200	1584	6936	1918	4308	3046	1018	6021	1446	9174	1092
Percentage of genes in orthogroups	96	92	93.8	96.3	87.6	91	98.5	82.5	98.3	81.9	97.2
Percentage of unassigned genes	4	8	6.2	3.7	12.4	9	1.5	17.5	1.7	18.1	2.8
Number of orthogroups containing species	13147	12777	17840	13594	15077	15201	14543	15341	14859	15139	13215
Percentage of orthogroups containing species	31.3	30.4	42.4	32.3	35.8	36.1	34.6	36.5	35.3	36	31.4
Number of species-specific orthogroups	356	145	4907	1869	447	529	826	529	1134	3006	855
Number of genes in species-specific orthogroups	1279	379	34643	10006	1469	2332	5778	1976	8509	13089	4137
Percentage of genes in species-specific orthogroups	4.2	1.9	30.8	19.2	4.2	6.9	8.3	5.7	10	25.8	10.8

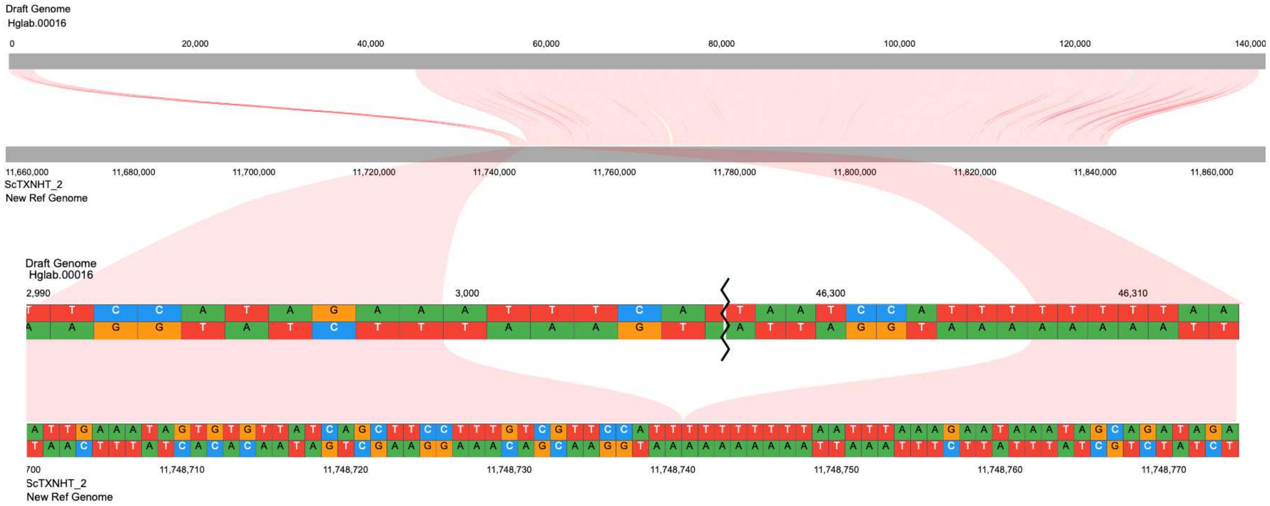

Figure S1. Example of genome correction performed to scaffold 2 of preliminary assembly using scaffolds from the draft genome of *H. glaberrima.*

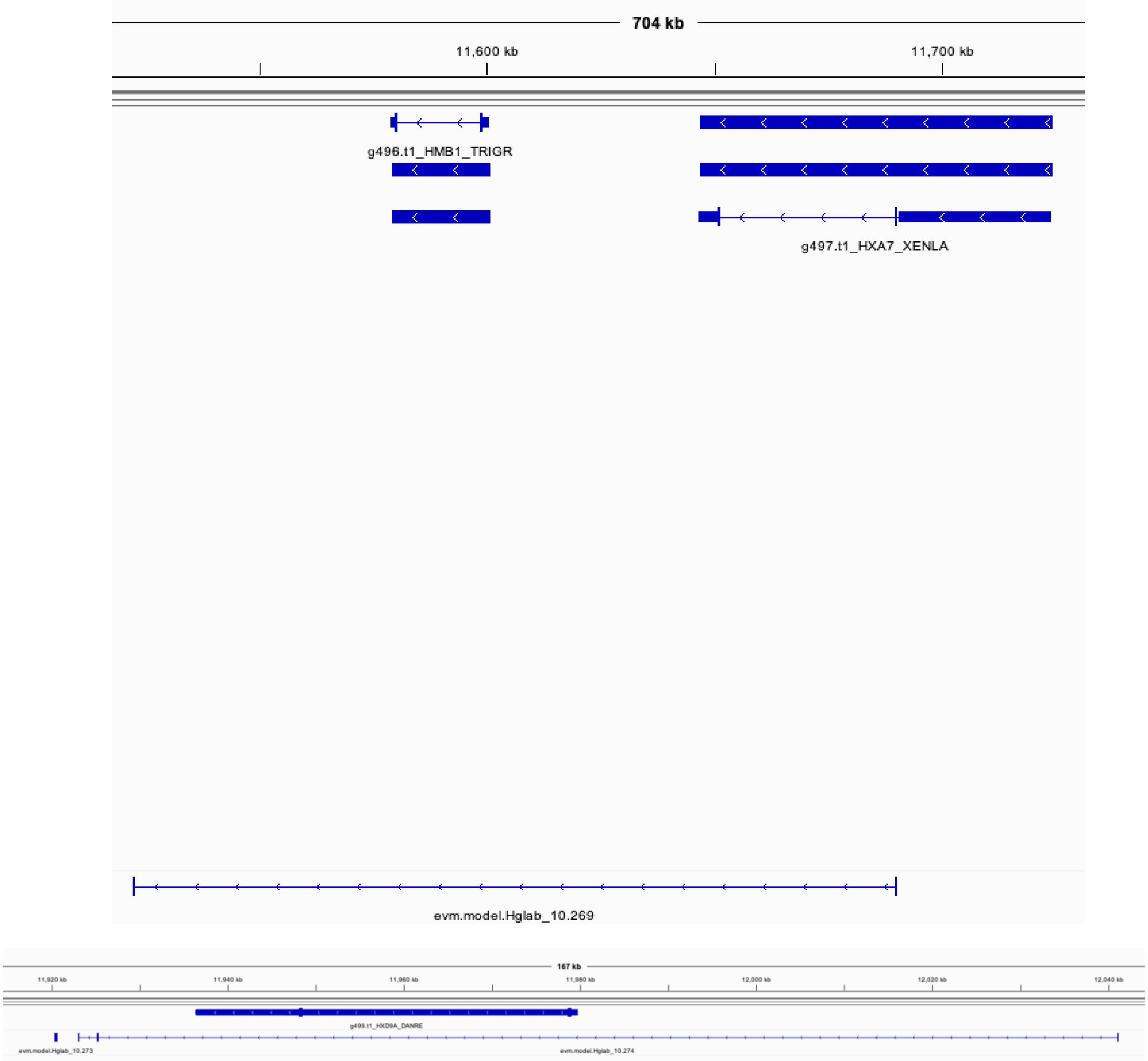

Figure S2. Example of regions of erroneous *Hox* gene models from EVM compared to the Augustus gene models.

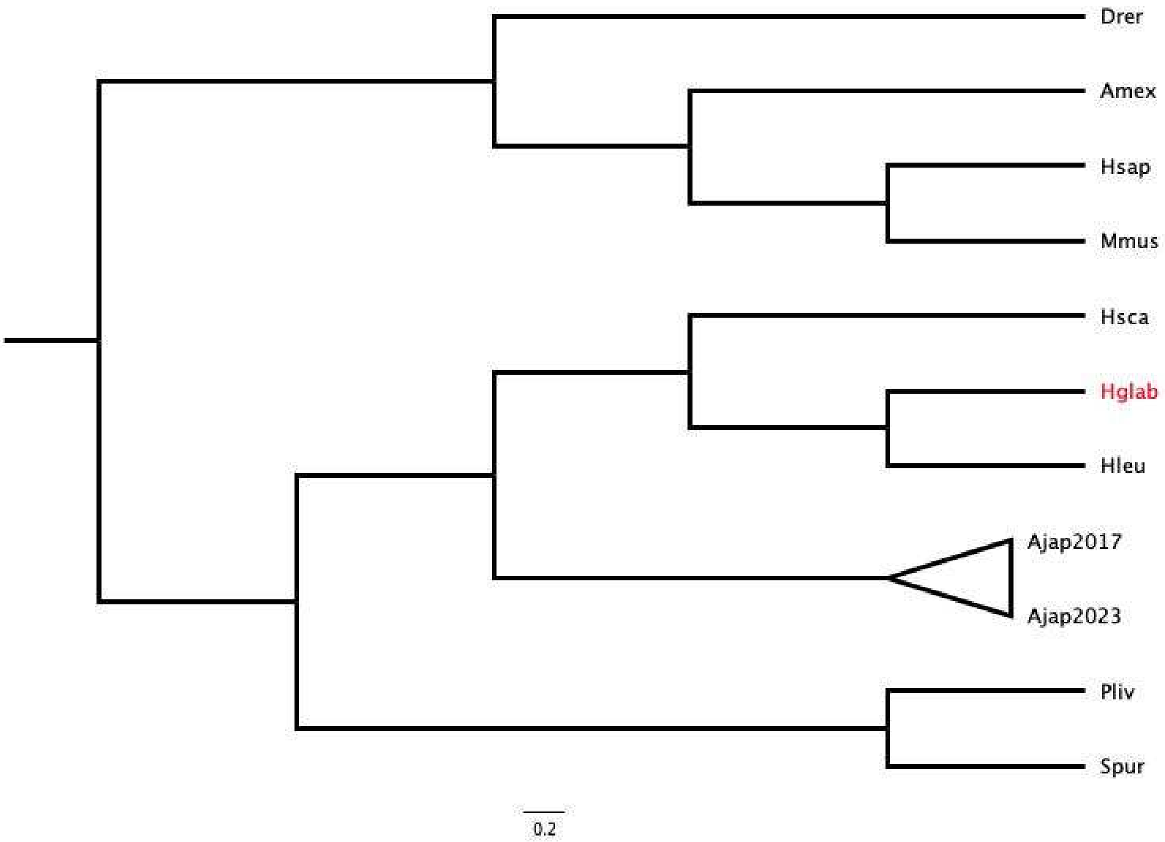

Figure S4. Species tree generated by OrthoFinder based on the protein model data sets provided per each species.

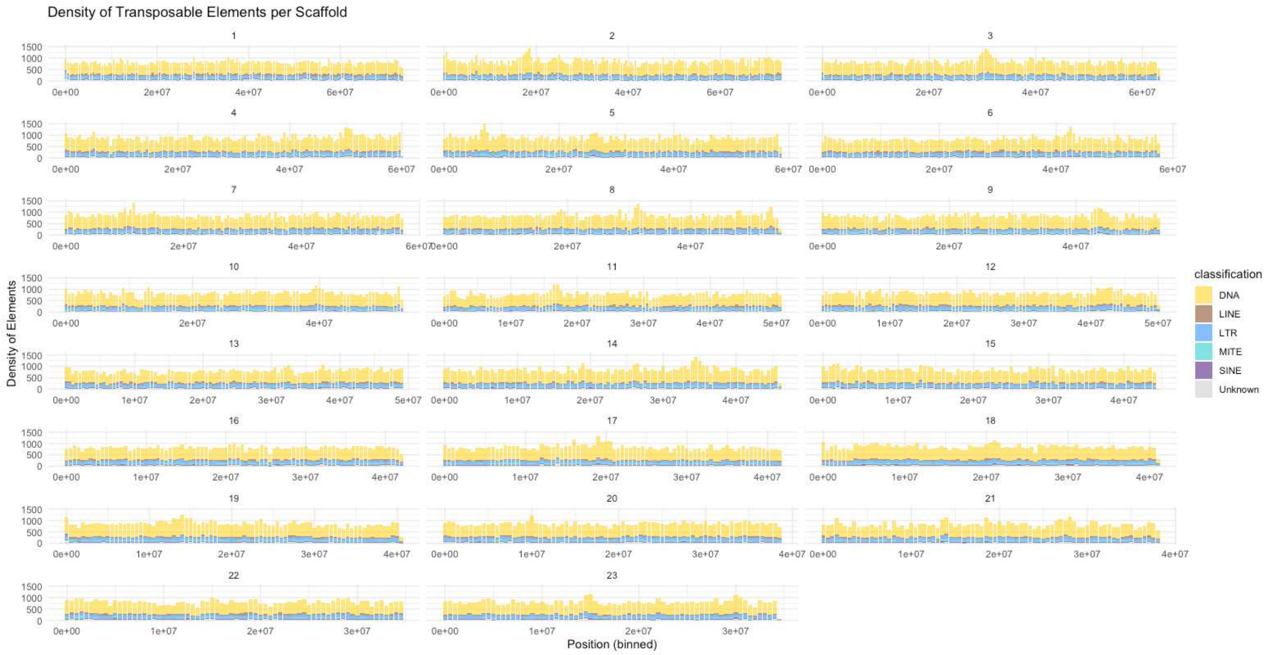

Figure S5. Density plot of the major TEs families across each of the chromosomal scaffolds of *H. glaberrima*.
